# The ATC12 small molecule inhibits the Aurora-A/TPX2 interaction and impairs the proliferation of breast cancer cells

**DOI:** 10.1038/s41419-026-08579-3

**Published:** 2026-03-24

**Authors:** Dalila Boi, Giulia Fianco, Federica Polverino, Francesco Fiorentino, Anna Mastrangelo, Simone Rossi, Elisabetta Rubini, Serena Rosignoli, Francesca Troilo, Maria Rosaria Antonelli, Dalila Tarquini, Laura Cervoni, Serena Rinaldo, Angela Tramonti, Eleonora Kristina Scarpone, Chiara Naro, Claudio Sette, Venturina Stagni, Gianni Colotti, Dante Rotili, Alessandro Paiardini, Giulia Guarguaglini, Italia Anna Asteriti

**Affiliations:** 1 Institute of Molecular Biology and Pathology, National Research Council of Italy, c/o Sapienza University of Rome, Rome, Italy; 2 Department of Biochemical Sciences, Sapienza University of Rome, Rome, Italy; 3 Centre for Regenerative Medicine “Stefano Ferrari”, Department of Life Sciences, University of Modena and Reggio Emilia, Modena, Italy; 4 GSTeP-Organoids Research Core Facility, IRCCS Fondazione Policlinico A. Gemelli, Rome, Italy; 5 Department of Neuroscience, Section of Human Anatomy, Catholic University of the Sacred Heart, Rome, Italy; 6 Istituto di Ricovero e Cura a Carattere Scientifico (IRCCS), Fondazione Santa Lucia, Signal transduction Unit, Rome, Italy; 7 Department of Science, Roma Tre University, Rome, Italy; 8 Biostructures and Biosystems National Institute (INBB), Rome, Italy

**Keywords:** Drug development, Mitosis

## Abstract

The Aurora-A kinase and its major regulator TPX2 act as key players during mitosis. Both are overexpressed in tumors, and the Aurora-A/TPX2 complex has been proposed as a potential oncogenic holoenzyme. Evidence of Aurora-A non-mitotic roles in cancer, some of which depend on its nuclear accumulation in interphase and are independent from the kinase activity, is emerging. Indeed, many Aurora-A ATP-competitive inhibitors have shown limited efficacy in clinical trials so far, highlighting the need for novel strategies to inhibit Aurora-A. Interestingly, our recent results suggest an involvement of TPX2 also in the non-mitotic protumorigenic roles of Aurora-A, which makes the Aurora-A/TPX2 complex a promising target. We previously described Aurora-A/TPX2 protein-protein interaction inhibitors. Here, starting from in silico analyses, we identified a new compound, i.e., ATC12, which we validated in vitro as a molecule able to bind Aurora-A and to compete with TPX2. We investigated the effects of ATC12 in 2D cultures and 3D mammospheres of breast cancer cell lines, as well as in patient-derived organoids, and observed an impairment of Aurora-A/TPX2 interaction and a decrease in cell viability and proliferation. Altogether, our observations support the targeting of the Aurora-A/TPX2 complex as a promising strategy for the development of novel anti-cancer therapeutics.

## Introduction

The serine/threonine kinase Aurora-A is a key mitotic regulator [1–4], with cell cycle-dependent levels that increase at the end of S phase and in G2 and peak in mitosis. Aurora-A mitotic roles include regulation of mitotic entry, centrosome maturation, and spindle organization. Aurora-A acts in mitosis through the phosphorylation of many substrates, and its activity is modulated by the concerted action of upstream regulators. A key step for ensuring the fully active conformation of Aurora-A is the interaction with TPX2, a microtubule-binding protein also required for Aurora-A stability and localization at the mitotic spindle [5–8].

Aurora-A is frequently overexpressed in many types of cancer, among which are breast, ovarian, prostate, and lung cancer [4, 9, 10]. Consequently, the kinase has been considered as a potential therapeutic target in the light of targeting mitosis to induce antiproliferative effects and cell death [11, 12]. Several compounds are therefore under evaluation in clinical trials as inhibitors of the kinase [10, 11, 13]. Among these, MLN8237 is considered the most specific Aurora-A inhibitor, and it has reached phase 3 for the evaluation of its effects in hematological tumors. Despite promising effects in preclinical studies, the results of clinical trials have not been exciting, in particular in patients with relapsed or refractory peripheral T-cell lymphoma [14, 15]. More promising results have been reported by performing combined treatments with canonical anti-cancer agents in solid tumors [MLN8237 and paclitaxel [16]].

A common feature of these inhibitors is that all bind to Aurora-A in the catalytic site and act as ATP-competitors. However, the ATP-binding site is highly conserved among kinases. Therefore, one of the critical issues of these compounds is the low selectivity of their action, with frequent off-target effects [17]. These issues have been the starting point for the research of new selective inhibitors of Aurora-A.

In recent years, the increasing knowledge about protein-protein interaction (PPI) interfaces has enabled the development of compounds capable of disrupting specific multi-protein complexes, and these are now considered promising tools for anti-cancer therapy [18, 19]. Regarding Aurora-A, given the key roles of TPX2 as an Aurora-A regulator, and the frequent co-overexpression of both proteins in cancer, the Aurora-A/TPX2 interaction has been investigated as a potential therapeutic target [20–22]. The first lead compounds capable of disrupting the Aurora-A/TPX2 interaction have been identified and validated both in vitro and in cell cultures, although the need for high concentrations of the compounds and their low solubility in the cell media remain limiting factors [23, 24]. Recently, Stockwell et al. have identified new compounds, one of which has also been tested in vivo, yielding promising results [25].

Interestingly, evidence accumulated in recent years about the interphase role of Aurora-A in cancer points toward non-mitotic and, in some cases, kinase-independent functions [26]. These oncogenic non-mitotic roles are linked to Aurora-A localization in interphase nuclei [27], providing additional value to Aurora-A PPI inhibitors. Given that the contribution of TPX2 to Aurora-A nuclear localization has been recently demonstrated [28], targeting the Aurora-A/TPX2 interaction looks promising, because PPI inhibitors are expected to affect also the non-kinase-related nuclear functions of Aurora-A, as compared to ATP-competitive inhibitors.

In this study, we optimized the search for compounds capable of interfering with the Aurora-A/TPX2 complex formation, building on our previously established workflow that led to the identification of our first Aurora-A/TPX2 PPI inhibitors [24]. Through a virtual screening approach, we identified a new compound, ATC12, which is capable of binding Aurora-A in vitro and competing with TPX2. Our results in breast cancer cells show that ATC12 treatment leads to a decrease in the Aurora-A/TPX2 interaction and influences cell viability and proliferation. Next, we confirmed the effects of ATC12 treatment on 3D cell culture systems of breast cancer cells (mammospheres). Of note, ATC12 was also effective in three patient-derived triple negative breast cancer organoids (TNBC PDOs), 3D models which better preserve the biological complexity and heterogeneity of original cancer tissues. Our results suggest that ATC12 may serve as a lead compound to broaden the spectrum of therapeutic tools targeting Aurora-A in cancer.

## Results

### Identification of molecules at the Aurora-A/TPX2 interaction interface

**Fig. 1:**
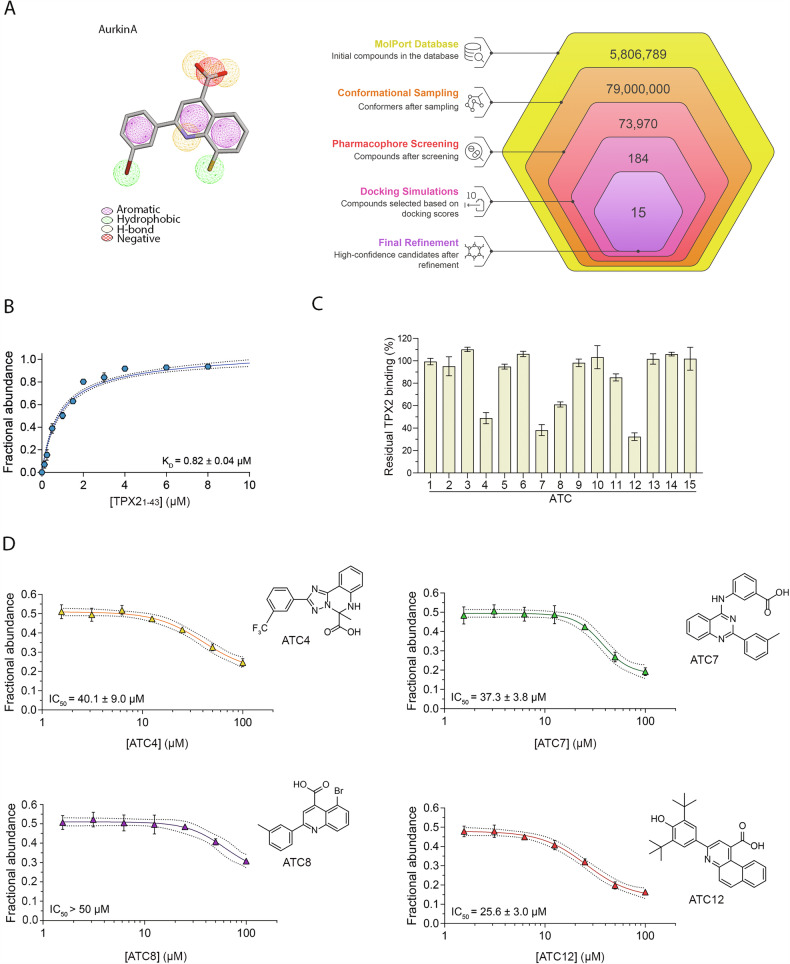
In vitro characterization of inhibitors of the Aurora-A/TPX2 interaction. **A** Pharmacophore map based on AurkinA and a flowchart illustrating the workflow used to identify the molecules derived from the pharmacophore hypothesis. **B** Native MS analysis of Aurora-A^KDCM^ (1 μM) incubated with increasing concentrations (0.125–12 μM) of TPX2_1-43_. The graph shows the plot of the fractional abundance of TPX2_1-43_-bound Aurora-A as a function of TPX2_1-43_ concentration, along with the relative fitting used to determine the apparent K_D_. **C** Native MS-based screening of small molecules as potential disruptors of the Aurora-A^KDCM^/TPX2_1-43_ interaction. All compounds were tested at 100 μM. The bar charts represent the residual TPX2_1-43_ binding to Aurora-A compared to control (0.5% DMSO). **D** Native MS analysis of Aurora-A^KDCM^/TPX2_1-43_ interaction in the presence of ATC4, ATC7, ATC8, and ATC12. The plot of TPX2_1-43_-bound Aurora-A fractional abundance as a function of compound concentration enables the quantification of the IC_50_ values. The relative Error bars indicate standard deviation (SD; *n* = 3). Dashed lines on the fitting represent “confidence bands” at a 95% confidence interval.

Using the pharmacophore hypothesis tailored from insights coming from the Aurora-A/TPX2 [6] and Aurora-A/AurkinA [23] complexes, we screened the purchasable MolPort compound library to identify molecules best matching critical interaction regions at the Aurora-A/TPX2 interface (Fig. 1A). This pharmacophore-based search, followed by docking studies, yielded an initial set of 184 molecules which emerged as promising candidates that effectively aligned with the hot spots of interaction identified in our previous work [24]. Candidate compounds were selected based on their ability to satisfy essential features within the pharmacophore model, with a strong emphasis on matching hydrophobic pockets, hydrogen bond donors, and acceptors critical to the Aurora-A/TPX2 binding cleft. This high-throughput selection process also enforced stringent spatial alignment with the active residues Tyr8 and Tyr10 of TPX2, ensuring interaction fidelity. Each compound’s binding pose was evaluated against the established pharmacophore constraints, resulting in a cohort of candidates predicted to exhibit stabilizing interactions at the Aurora-A interface. Among these 184 molecules, further docking analysis highlighted a subset of 15 top-scoring compounds that consistently scored high across all docking and pharmacophore criteria (Fig. 1A and Supplementary Table I).

To further narrow down the cohort of lead compounds, the top 15 scoring molecules were tested for their ability to disrupt the Aurora-A/TPX2 complex in vitro. Using native mass spectrometry (nMS), we assessed the displacement of the TPX2_1-43_ peptide fragment (i.e., the TPX2 binding region for Aurora-A) from the C290A/C393A catalytic domain of Aurora-A (Aurora-A^KDCM^), which was chosen as it is reported to be more stable for in vitro analysis [29]. TPX2_1-43_ demonstrated strong affinity for Aurora-A (K_D_ 0.82 µM, Figs. 1B and S1A), supporting the reliability of the used experimental conditions. Each compound (100 µM) was then incubated with Aurora-A^KDCM^ (1 µM) and TPX2_1-43_ (1 µM) for 10 min at room temperature and analyzed by nMS. Of all the tested compounds, four (ATC4, ATC7, ATC8, and ATC12) were able to reduce the abundance of the Aurora-A/TPX2_1-43_ bound peak by 40% or higher (Fig. 1C).

The compounds identified in the competition experiments were further tested to assess their potency in disrupting the interaction between TPX2 and Aurora-A. Each compound was titrated (1.56, 3.12, 6.25, 12.5, 25, 50, and 100 µM) under the same experimental conditions described above, and the IC_50_ values were determined as detailed in the “Materials and methods” section (Fig. 1D). AurkinA served as a reference control and exhibited an IC_50_ value consistent with the one reported in literature [Fig. S1B[23],]. As shown in Figs. 1D and S1C, the ATC7 and ATC12 compounds demonstrated the highest potency in disrupting the Aurora-A/TPX2 interaction, with IC_50_ values of 37.3 µM and 25.6 µM, respectively, and were selected for further analysis.

### ATC7 and ATC12 interact with Aurora-A without inhibiting its activity in vitro

**Fig. 2:**
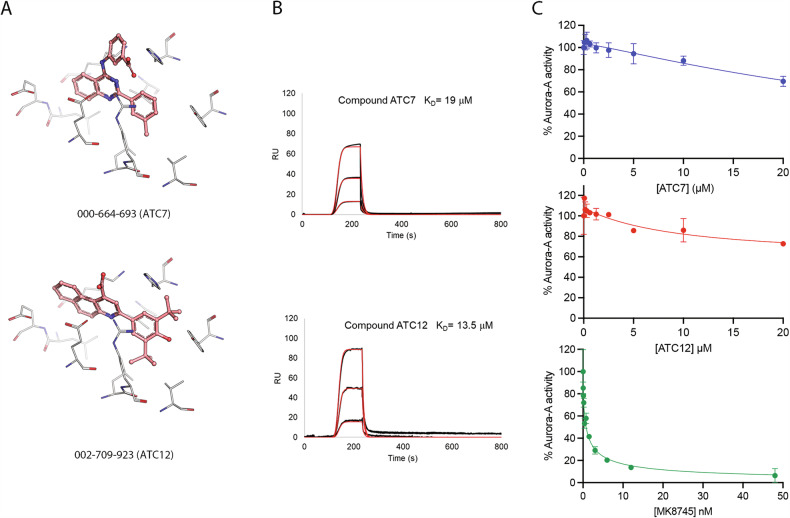
In vitro binding of ATC molecules to Aurora-A and kinase activity evaluation. **A** Top-scored docking poses of ATC7 and ATC12. Ligands (pink sticks) are shown in their predicted binding conformations within the Y-pocket, for which representative residues are shown in sticks (light gray). **B** OneStep sensorgrams showing the interaction of the ligand Aurora-A^KDCM^, immobilized onto a HisCap Sensor Chip, with analytes (ATC7 and ATC12) injected at different concentrations, i.e., 3.7 μM, 11 μM, and 33 μM. In all experiments, the increase in RU relative to baseline indicates complex formation between the immobilized Aurora-A ligand and the analytes. The plateau region represents the steady-state phase of the interaction. The decrease in RU after 240 s indicates analyte dissociation from the immobilized Aurora-A after buffer injection. Overall kinetic evaluation of the 3 sensorgrams (black curves) was carried out as 1:1 ligand-analyte interactions; fittings are shown as thin red curves; K_D_ values are indicated for each compound. **C** Kinase activity assay of the Aurora-A^KDCM^ in the presence of increasing concentrations of ATC7 and ATC12. The inhibition curve obtained using the ATP-competitive inhibitor MK8745 is shown as a positive control. The graphs show the activity of pre-phosphorylated Aurora-A as relative light units (RLU) with respect to the control condition (100%, DMSO). One of two independent experiments is shown; each data point represents the average value of two technical replicates. Standard errors are shown.

To validate the predicted affinity of ATC7 and ATC12 according to their docking poses (Figs. 2A, S2A) and to better delve into the ability of our selected compounds to disrupt the Aurora-A/TPX2 complex in vitro, we assessed the thermodynamic parameters of the interaction between Aurora-A^KDCM^ and ATC7 or ATC12 through Surface Plasmon Resonance (SPR) (Fig. 2B). The measured K_D_ values were in the low micromolar range for both ATC7 and ATC12 (19 µM and 13.5 μM, respectively), indicating good binding affinity between the compounds and the protein. As a positive control, the previously described Aurora-A allosteric inhibitor AurkinA displayed a K_D_ of 10.2 μM (Fig. S2B). Similar results were obtained in Isothermal Titration Calorimetry (ITC) experiments, using the catalytically inactive Aurora-A^KDCM^ D274N mutant (Fig. S2C).

To test the allosteric behavior of ATC7 and ATC12, we performed an ADP-Glo™ Kinase Assay, which enabled us to evaluate the ability of Aurora-A^KDCM^ to phosphorylate the Myelin Basic Protein (MBP) (Fig. 2C), in the presence of increasing concentrations of ATC7 or ATC12 (from 0.075 µM to 20 µM). Differently from results obtained with the known Aurora-A kinase inhibitor MK8754, ATC7 and ATC12 did not decrease the enzymatic activity of the kinase.

Taken together, these data indicate that ATC7 and ATC12 can bind Aurora-A in vitro and disrupt the formation of the Aurora-A/TPX2 complex, without impairing the catalytic activity of Aurora-A.

### ATC7 and ATC12 modulate the Aurora-A/TPX2 complex in cultured cancer cells

**Fig. 3:**
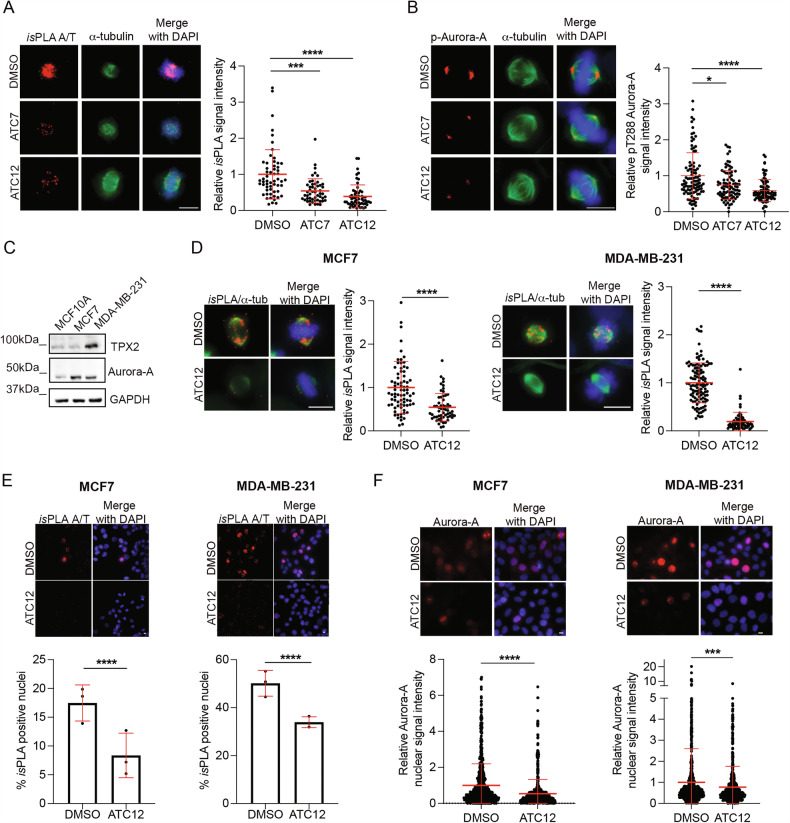
ATC12 impairs Aurora-A/TPX2 complex formation in cancer cells. Mitotic Aurora-A/TPX2 (A/T) *is*PLA signals in U2OS (**A**), MCF7 (**D**, left), or MDA-MB-231 (**D**, right) under the indicated treatments are shown in the fluorescence panels and quantified in the dot plots. **B** Representative IF images of pThr288 Aurora-A in control (DMSO) and ATC7- or ATC12-treated prometa-metaphases (U2OS). pThr288 Aurora-A signal intensity at each spindle pole is quantified in the dot plot on the right. **C** Immunoblotting with TPX2 and Aurora-A antibodies in total lysates of MCF10A, MCF7, and MDA-MB-231 cultures. GAPDH was used as a loading control. **E** MCF7 and MDA-MB-231 cells positive for nuclear Aurora-A/TPX2 *is*PLA signals are shown in the fluorescence panels and quantified in the histograms below. **F** Aurora-A signals are shown in the IF panels and quantified in the dot plots below. For dot plots, each dot represents the fluorescence value of single cells within selected regions: interphase nuclei (**F**), prometa-metaphase spindles (**A**, **D**), or spindle poles (**B**). The control condition is set as 1 in relative quantifications. Sample size per condition: DMSO: 57 (**A**), 100 (**B**), 77 (**D**, MCF7), 114 (**D**, MDA-MB-231), 1183 (**E**, MCF7), 662 (**E**, MDA-MB-231), 880 (**F**, MCF7), 891 (**F**, MDA-MB-231); ATC7: 51 (**A**), 87 (**B**); ATC12: 58 (**A**), 84 (**B**), 58 (**D**, MCF7), 67 (**D**, MDA-MB-231), 777 (**E**, MCF7), 1188 (**E**, MDA-MB-231), 809 (**F**, MCF7), 745 (**F**, MDA-MB-231); from 2 (**A**, **B**) or 3 (**D**–**F**) independent experiments. Error bars: SD; *p < 0.05; ***p < 0.001; ****p < 0.0001; Kruskal–Wallis test (**A**, **B**), Unpaired *t*-test (**D**), chi-square (and Fisher’s exact) test (**E**), and Mann–Whitney test (**F**). Scale bars, 10 μm.

The promising results obtained in vitro encouraged us to evaluate the activity of ATC7 and ATC12 in cultured cells. To assess the ability of the compounds to impair the Aurora-A/TPX2 interaction in cells, we analyzed the complex formation at the mitotic spindle, where the two proteins are known to interact. In situ Proximity Ligation Assays (*is*PLA) were performed in osteosarcoma U2OS cells. The administration of 10 μM ATC7 or ATC12 for 24 h leads to a significant decrease in Aurora-A/TPX2 *is*PLA signal intensity at the mitotic spindle in prometa- and metaphases, with a stronger effect observed after ATC12 treatment (about 61% and 46% decrease of *is*PLA signal in ATC12 or ATC7 treated samples, respectively; Fig. 3A). Given the importance of TPX2 for Aurora-A kinase activation, we evaluated under the same conditions the extent of pT288 Aurora-A auto-phosphorylation by western blot (WB) and immunofluorescence (IF). While both treatments were effective in reducing pT288 Aurora-A by WB (Fig. S3A), single cell analysis revealed that, consistent with *is*PLA results, ATC12-induced reduction in the phosphorylation of Aurora-A-T288 at spindle poles was more significant compared to both ATC7 (Fig. 3B) or AurkinA (Fig. S3B) treatments.

Results obtained in U2OS cells prompted us to focus on the most effective ATC12 compound. We moved to breast cancer cell lines, based on the notion that Aurora-A and TPX2 are frequently co-overexpressed in breast cancer [9, 12, 27, 30]. The two used cell lines are representative of different cellular models: MCF7 cells are poorly-aggressive and non-invasive ER+, luminal A and TP53 wild-type [31, 32], whereas MDA-MB-231 cells are basal-like, triple negative (ER-, PR- and HER2-) and TP53 mutated, which makes them highly aggressive [31, 33, 34].

WB experiments show that levels of Aurora-A are comparable in the two cell lines and are higher than those observed in MCF10A nontransformed breast epithelial cells (Fig. 3C); TPX2 protein levels were instead higher in MDA-MB-231 cells compared to both MCF7 and MCF10A (Fig. 3C).

As for U2OS, the Aurora-A/TPX2 interaction decreases in mitotic cells at the spindle region after ATC12 treatment (*is*PLA signal intensity decreases by 50% and 81% in MCF7 and MDA-MB-231, respectively; Fig. 3D). IF experiments also showed a decrease of total Aurora-A signal intensity at mitotic spindle poles (reduction of 50% in MCF7 and about 25% in MDA-MB-231 cells; Fig. S3C). This observation is consistent with Aurora-A/TPX2 complex impairment, since the interaction with TPX2 is responsible for Aurora-A localization at spindle microtubules and is important for Aurora-A protein stability [5, 8].

Based on recent evidence highlighting oncogenic roles of nuclear Aurora-A during interphase [27, 35] and the involvement of TPX2 in Aurora-A nuclear accumulation [28], we evaluated the effects of ATC12 treatment at the nuclear level during interphase. Notably, the percentage of positive nuclei for Aurora-A/TPX2 *is*PLA signal decreased in both cell lines (from 17.5% to 8.4% in MCF7 and from 50% to 34% in MDA-MB-231; Fig. 3E), confirming that ATC12 disrupts the Aurora-A/TPX2 interaction also in interphase nuclei. Interestingly, a parallel reduction of the IF Aurora-A nuclear signal intensity was observed (by 47% in MCF7 and 22% in MDA-MB-231; Fig. 3F).

Altogether, these results indicate that ATC12 can interfere with the Aurora-A/TPX2 interaction in both interphase and mitosis in cancer cell lines.

### Disruption of the Aurora-A/TPX2 interaction influences cell growth and viability

**Fig. 4:**
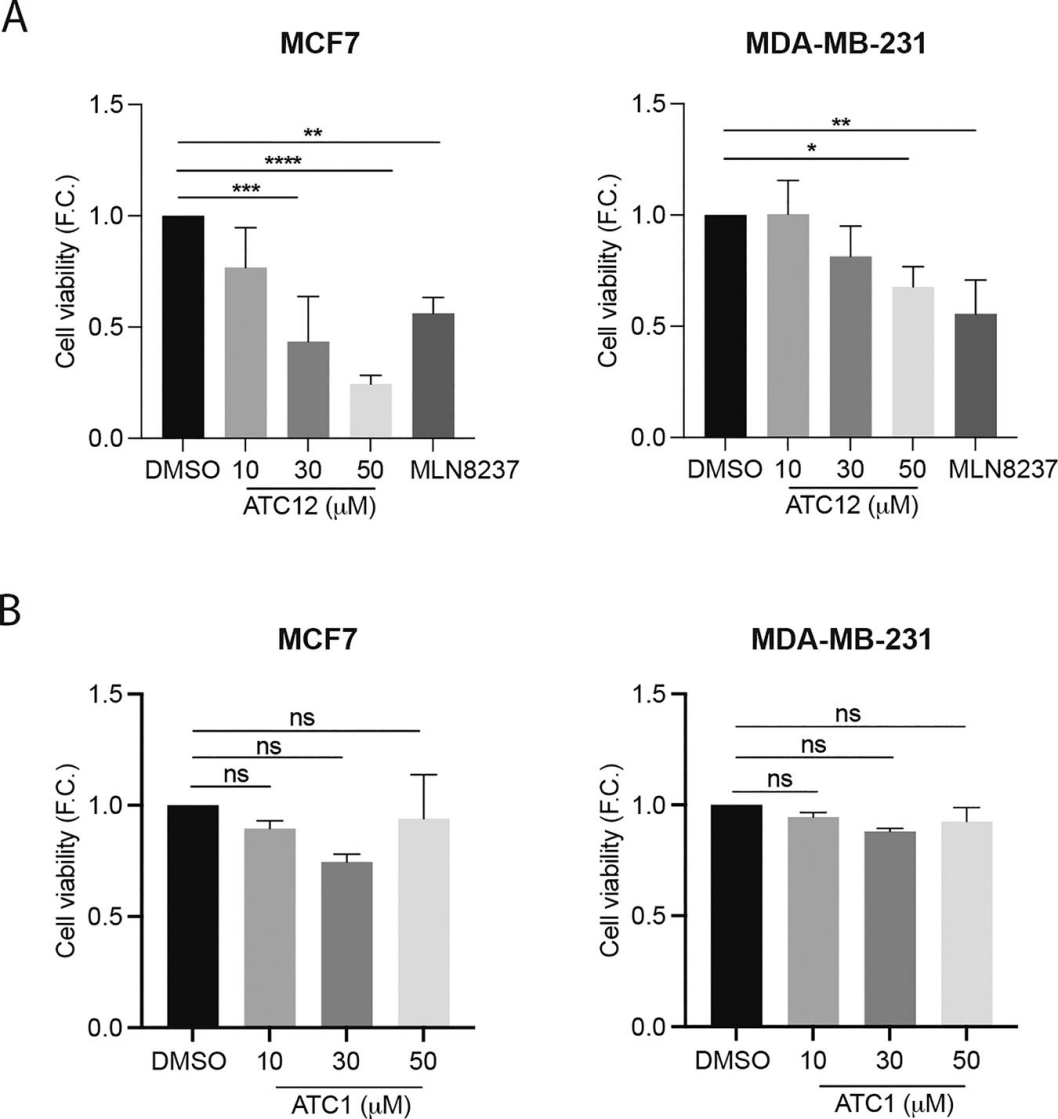
ATC12 influences the viability of breast cancer cells. MTT assays of MCF7 (left) and MDA-MB-231 (right) cells treated with 1 μM of MLN8237, or ATC12 (**A**) and ATC1 (**B**) at the indicated concentrations for 72 h, in technical sextuplicate from 3 (**A**) or 2 (**B**) independent experiments. Fold changes (F.C.) are shown with respect to the control condition, set as 1. Error bars: SD (**A**) or SEM (**B**); *p < 0.05; **p < 0.01; ***p < 0.001; ****p < 0.0001; ns not significant, Ordinary One-way ANOVA multiple comparisons test.

Following confirmation that ATC12 induces the disruption of the Aurora-A/TPX2 interaction in breast cancer cells, we sought to evaluate its functional effects on cell viability by performing dose-response MTT assays after 72 h of treatment. A difference in the response to the treatment was observed in MCF7 compared to MDA-MB-231 cells (Fig. 4A). Specifically, MCF7 cell viability significantly decreases in a dose-dependent manner, starting from 30 μM ATC12. MDA-MB-231 cells are more resistant to the treatment, with a small, but significant decrease in cell viability only when treated with 50 μM ATC12. For all MTT assays, MLN8237 (1 μM) was used as positive control and the well-known effects of this ATP-competitive Aurora-A inhibitor on cell viability were confirmed (Fig. 4A). To verify the specificity of the effect of ATC12 on cell viability, we performed MTT assays with ATC1, a compound that could not efficiently disrupt Aurora-A/TPX2 binding according to our nMS experiments (Fig. 1C). ATC1 treatment was performed under the same experimental conditions used for ATC12 and no significant decrease in cell viability was observed in the two cell lines (Fig. 4B). These results indicate that ATC12 can specifically affect cell viability in breast cancer cell lines.

**Fig. 5:**
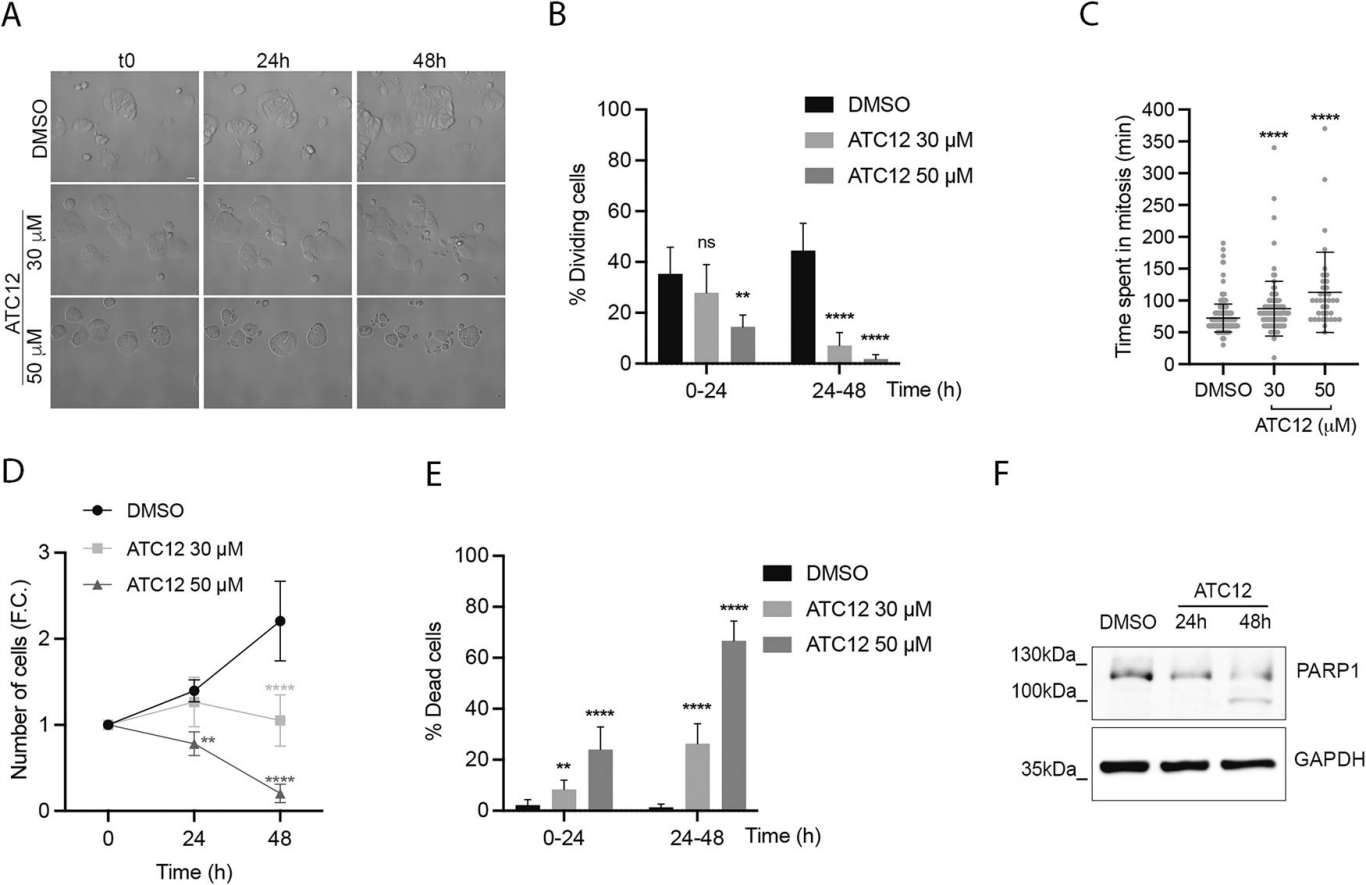
ATC12 treatment impairs proliferation and induces cell death in MCF7 cultures. **A** Representative images of time-lapse video recording of MCF7 cells treated with DMSO, 30 or 50 μM ATC12 for 48 h, acquired at t0, 24, and 48 h. The percentage of dividing cells within 24 h intervals is shown in (**B**), while the time spent in mitosis (minutes from the round-up to the complete re-attachment of daughter cells) is shown in (**C**). The graph in (**D**) indicates the increase (F.C., fold change) in cells at 24 and 48 h, with respect to the initial number of cells (t0) per condition. **E** Histograms represent the percentage of dead cells within 24 h intervals. Sample size per condition, from three independent experiments: DMSO: 240 (**B**, **D**, **E**), 237 (**C**); ATC12 30 μM: 271 (**B**, **D**, **E**), 96 (**C**); ATC12 50 μM: 209 (**B**, **D**, **E**), 37 (**C**). **F** Immunoblotting with PARP antibody of MCF7 total cell lysates upon 24 or 48 h treatment with 50 μM ATC12. GAPDH was used as a loading control. Error bars: SD; **p < 0.01, ****p < 0.0001; ns not significant, Fisher’s exact test (**B**, **E**), Kruskal–Wallis test (**C**), Two-way ANOVA multiple comparisons test (**D**). Scale bar, 20 μm.

In order to clarify the underlying mechanisms, we evaluated cell proliferation by time-lapse microscopy in MCF7 and MDA-MB-231 cells treated with 30 and 50 μM ATC12. In the MCF7 cell line, the dividing cells were counted within two time intervals (t0–24 h and 24–48 h; Fig. 5A, B), based on toxicity assays indicating a strong viability reduction at 48 h with significantly lower effects in nontransformed MCF10A cells (Fig. S4A). A significant decrease of the percentage of cells entering mitosis in the first 24 h of treatment (from 35.3% in control conditions to 14.4% in cultures treated with 50 μM ATC12) was observed, and the effect was exacerbated when cells were analyzed from 24 to 48 h of treatment (from 44.5% in control conditions to 7% and 1.8% in 30 and 50 μM ATC12-treated samples, respectively). In addition, cells that entered mitosis within the 48 h of ATC12 treatment required an extended time, compared to control cells, to conclude the mitotic process, in particular at the highest concentration (average duration of 87 min in 30 μM and 113 min in 50 μM, compared to 72 min in control cultures; Fig. 5C). By analyzing the number of cells throughout the 48 h of treatment, we observed a strong impairment of cell growth in the presence of ATC12, compared to control cultures (Fig. 5D), with no major effects on cell cycle distribution as assessed by parallel FACS analyses (Fig. S4B). Indeed, the number of cells decreased over time, and this was accompanied by massive cell death (Fig. 5E; 8.4 and 24% within the first 24 h, 26 and 67% after 48 h for 30 and 50 μM ATC12, respectively). The induction of cell death was confirmed by WB analysis on lysates from cell cultures treated with ATC12 for 48 h, which shows the appearance of cleaved PARP1 (Fig. 5F). This observation suggests that ATC12 treatment induces apoptosis in MCF7 cells.

**Fig. 6:**
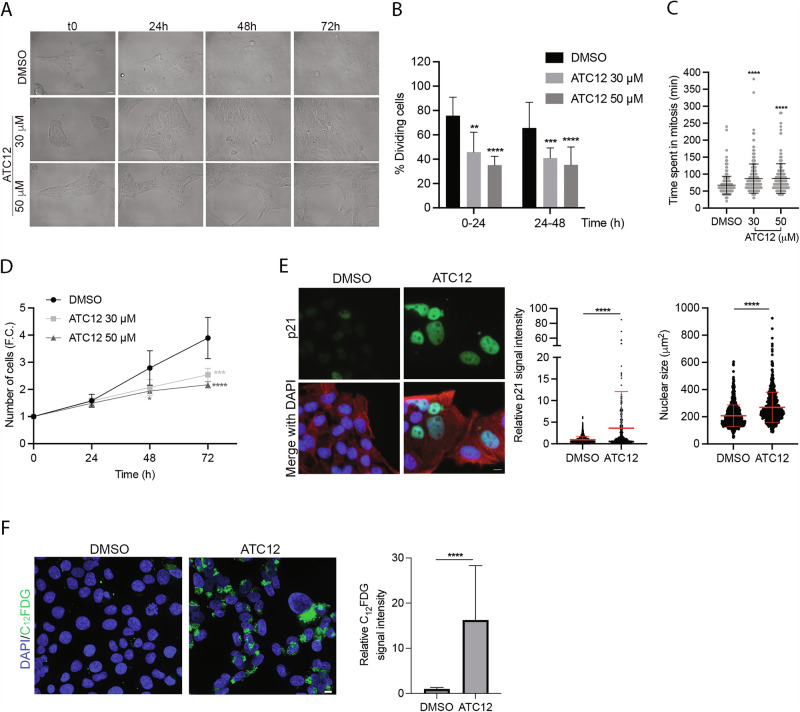
ATC12 treatment impairs proliferation and induces senescence in MDA-MB-231 cultures. **A** Representative images of time-lapse video recording of MDA-MB-231 cells treated with DMSO, 30 or 50 μM ATC12 for 72 h, acquired at t0, 24, 48, and 72 h. The percentage of dividing cells within 24 h intervals is shown in (**B**), while the time spent in mitosis (minutes from the round-up to the complete re-attachment of daughter cells) is shown in (**C**). The graph in (**D**) indicates the increase (F.C., fold change) in cells at 24, 48, and 72 h, with respect to the initial number of cells (t0) per condition. Sample size per condition, from two (ATC12 30 μM) or three (DMSO and ATC12 50 μM) independent experiments: DMSO: 231 (**B**, **D**), 356 (**C**); ATC12 30 μM: 255 (**B**, **D**), 261 (**C**); ATC12 50 μM: 221 (**B**, **D**), 190 (**C**). **E** Representative IF images of p21 staining in MDA-MB-231 cells treated with 50 μM ATC12 for 72 h. The graphs show: quantification of p21 nuclear signal intensity (left, DMSO: 1051 cells; ATC12: 415 cells, from three independent experiments) and the nuclear size (right, DMSO: 829 cells; ATC12: 587 cells, from three independent experiments). **F** Representative panels of C_12_FDG staining in MDA-MB-231 cells treated with 50 μM ATC12 for 72 h. C_12_FDG signal intensity per field (DMSO: 1919 cells; ATC12: 1058 cells, from three independent experiments) is quantified in the graph. The control condition is set as 1 in relative quantifications. Error bars: SD; *p < 0.05; **p < 0.01; ***p < 0.001; ****p < 0.0001; Fisher’s exact test (**B**), Kruskal–Wallis test (**C**), Two-way ANOVA multiple comparisons test (**D**), Mann–Whitney test (**E**, **F**). Scale bars, 20 μm (**A**); 10 μm (**E**, **F**).

Similar analyses carried out in MDA-MB-231 cells (Fig. 6A) showed a dose-dependent decrease in the percentage of dividing cells in both the 0–24 and 24–48 h intervals from ATC12 treatment and an increase in the time spent in mitosis (Fig. 6B, C). We also confirmed a dose-dependent decrease in cell proliferation, in this case appearing from 48 h on for 50 μM ATC12 and particularly evident after 72 h of treatment (Fig. 6D). Interestingly, analysis of the 72 h video recording and of PARP1 by WB (Fig. S4C, D) showed no cell death induction in this cell line, also at highest ATC12 tested concentration.

The reduced cell proliferation without cell death induction observed in MDA-MB-231 cells suggested that ATC12 treatment may influence cell cycle progression. We therefore decided to analyze the levels of p21, a negative cell cycle regulator. Cells were treated with 50 μM ATC12 for 72 h, and p21 levels were evaluated by IF experiments (Fig. 6E). The analysis revealed a significant increase in p21 signal intensity after ATC12 treatment (about 3.5-fold) compared to control interphases. We also noticed the appearance of cells with large nuclei in ATC12-treated MDA-MB-231 cultures. Indeed, the average size of interphase nuclei after 72 h of ATC12 treatment, measured based on DAPI staining (Fig. 6E), indicated a significant increase (average area: 270 μm^2^) compared to control cells (average area: 207.5 μm^2^). p21 levels combined with nuclear size increase were suggestive of senescence induction. To explore this hypothesis, we performed senescence assays using the C_12_FDG, a lipophilic green fluorescent substrate for β-galactosidase detection. The significant increase (16-fold) of C_12_FDG fluorescence signal intensity after 72 h of 50 μM ATC12 treatment confirmed the induction of senescence (Fig. 6F). Supporting the induction of cell death as the main response to ATC12 in MCF7 cells, no increase in nuclear area or senescence was observed (Fig. S4E, F).

Altogether, these experiments indicate that interfering with Aurora-A/TPX2 complex formation by ATC12 impairs the proliferation of MCF7 and MDA-MB-231 cells, triggering a cell death or a cell cycle arrest/senescence response, respectively.

### Evaluation of ATC12 effects in 3D cultures of breast cancer cells

**Fig. 7:**
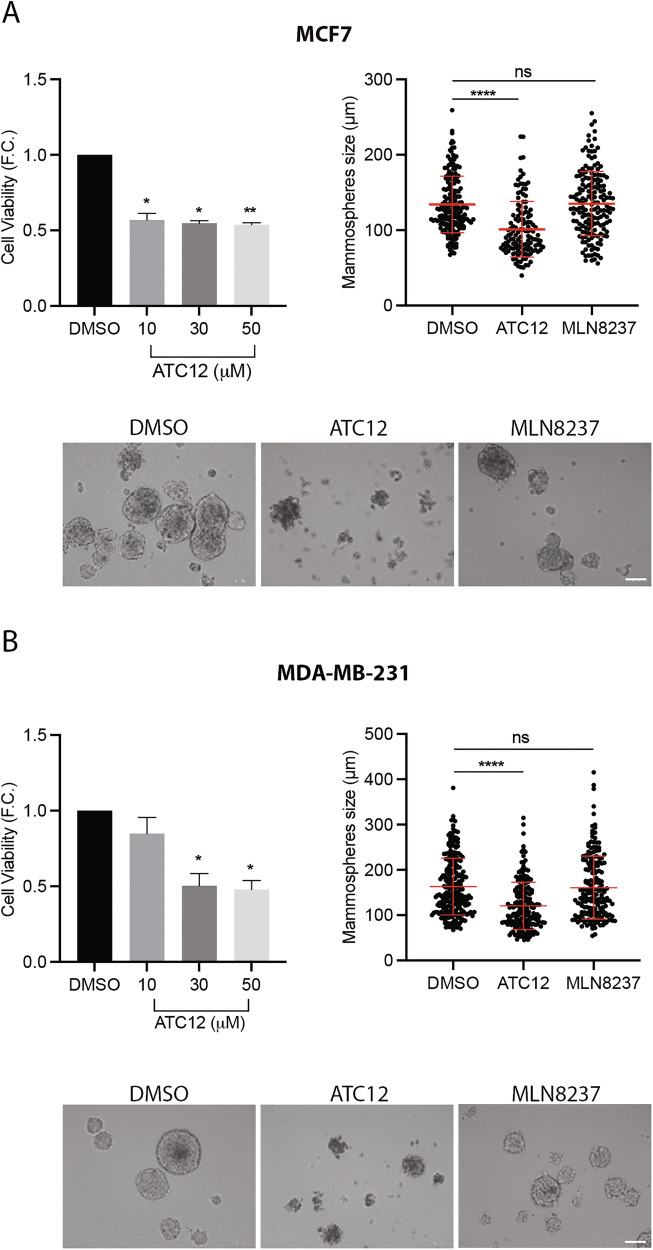
Effects of ATC12 treatment in MCF7 and MDA-MB-231 cells grown as mammospheres. Histograms on the left show the viability of mammospheres, derived from MCF7 (**A**) and MDA-MB-231 (**B**), under the indicated conditions, from MTS assays in technical sextuplicate; two independent experiments. Fold changes (F.C.) are shown with respect to the control condition, set as 1. The dot plots on the right represent the quantification of the size of mammospheres derived from MCF7 (**A**) and MDA-MB-231 (**B**) cells from two independent experiments. Sample size per condition: DMSO: 184 (**A**) and 247 (**B**); ATC12: 132 (**A**) and 193 (**B**); MLN8237: 169 (**A**) and 185 (**B**). Representative images of mammospheres are shown below. Error bars: SEM (histograms) or SD (dot plots); *p < 0.05; **p < 0.01; ****p < 0.0001; ns not significant; One-way ANOVA (histograms) and Kruskal–Wallis (dot plots) multiple comparisons test. Scale bars, 100 μm.

In order to further evaluate the functional consequences of ATC12 treatment on breast cancer cells, we moved to 3D culture conditions (Fig. 7), offering a more physiologically relevant model compared to traditional 2D cultures. MCF7 and MDA-MB-231 cells were seeded in low attachment plates to generate mammospheres; after 7 days of mammosphere growth, followed by 72 h of treatment with increasing doses of ATC12 (10, 30, and 50 μM), MTS assays were performed to evaluate mammosphere viability. In MCF7, a significant decrease in cell viability was evident starting from 10 μM, while in MDA-MB-231, 30 μM ATC12 was required (Fig. 7A, B; histograms on the left). Interestingly, the effects of ATC12 treatment on MDA-MB-231 cell viability were stronger in mammospheres compared to those observed in 2D cultures (Fig. 4A).

We also measured the size of mammospheres after 4 days of growth, followed by 48 h of treatment with ATC12 (50 μM). A significant decrease (roughly 25% reduction) was observed in mammospheres derived from both cell lines (Fig. 7A, B; dot plots on the right). Notably, treatment with MLN8237 (1 μM) did not significantly influence the size of mammospheres, suggesting that this effect is not dependent on Aurora-A kinase activity.

Together, these results indicate that disrupting the Aurora-A/TPX2 complex is an effective strategy to reduce the ability of breast cancer cells to grow as mammospheres.

### ATC12 reduces the viability of TNBC BCOs

**Fig. 8:**
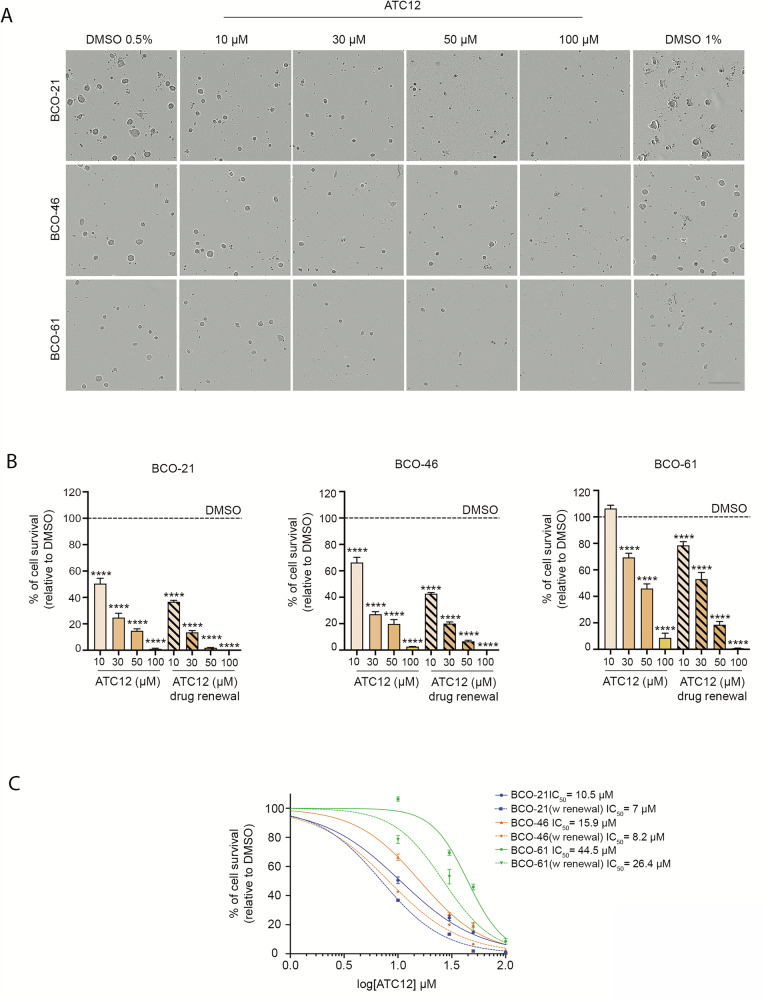
Effects of ATC12 treatment in patient-derived organoids (PDOs). **A** Representative micrographs of BCO-21, BCO-46, and BCO-61 continuously treated with ATC12 for 5 days, as indicated. Scale bar, 200 µm. **B** Bar graph illustrating the survival percentage of PDOs treated with ATC12, either continuously or with a drug renewal at half-time of the treatment. Every treatment has been normalized to its relative DMSO control. Error bars: SD (*n* = 3). ****p < 0.0001; One-way ANOVA test. **C** Dose-response curves of BCO-21, BCO-46, and BCO-61 treated with ATC12, either continuously or with a drug renewal at half the time of the treatment. The fitted curves were used to extrapolate IC_50_ values.

To validate ATC12 effects in breast cancer models that are representative of the actual disease, we decided to use breast cancer organoids (BCOs), developed from chemo-naïve biopsies of TNBC patients that were refractory to cytotoxic chemotherapy [36, 37]. Three BCOs (BCO-21, -46, -61; Supplementary Table II) were used, in which we preliminarily assessed sensitivity to Aurora-A inhibition by MLN8237 treatment (Fig. S5A, B). BCOs were then treated with increasing concentrations of ATC12 for 5 days, and viability was evaluated (Fig. 8). In all BCOs, viability was significantly impaired in a dose-response manner (Fig. 8A, B). The calculated IC_50_ for BCO-21 and -46 was around 10–15 μM, while BCO-61 required a higher concentration for achieving an effective response (Fig. 8C). Interestingly, in all cases, the effects of ATC12 were increased by re-adding the compound halfway through the treatment, suggesting a potential issue of stability in the long term (Figs. 8B, C and S5C). These results indicate that inhibiting the Aurora-A/TPX2 complex formation is effective in reducing the viability of complex and heterogeneous TNBC models, thus representing a promising chemotherapeutic strategy with the potential to be used either alternatively or adjunctively with existing therapies, particularly in settings of chemoresistance.

## Discussion

The Aurora-A kinase is a key regulator of mitosis, often overexpressed in cancer. In recent years, a plethora of cancer-associated non-mitotic functions of Aurora-A have been described [26, 27], which do not always require Aurora-A activity, while more likely relying on its interactions with partners, including oncogenes (e.g., Myc or FOXM1[35, 38]) or activators (i.e., TPX2[28]), pointing out that the inhibition of the kinase activity is no longer the unique path to pursue. In particular, the Aurora-A/TPX2 complex has been proposed as an oncogenic holoenzyme, given the frequent co-overexpression of the two proteins in tumors. This observation provides a rationale for considering the complex as a potential therapeutic target [20, 22]. Targeting the Aurora-A/TPX2 complex with PPI inhibitors is envisaged as a promising approach to counteract the oncogenic roles of the holoenzyme and overcome the critical issues exhibited by the ATP-competitive inhibitors of Aurora-A in clinical trials [[13, 15]; ClinicalTrials.gov]. As the PPI surfaces they target are more distinctive and context-specific than the highly conserved active sites of kinases, PPI inhibitors can reduce cytotoxic effects from off-target inhibition and allow selective targeting of specific pools of the protein of interest, which may play different roles in normal versus oncogenic processes [39].

In this work, we describe the validation of the ATC12 compound, a new inhibitor of the Aurora-A/TPX2 complex, identified starting from previously described molecules C20 and C23 [24] and AurkinA [23]. Starting from a dataset of potential Aurora-A/TPX2 inhibitors, we selected the two most promising compounds (i.e., ATC7 and ATC12) for further characterization in cellular assays. Preliminary results obtained in the U2OS osteosarcoma cell line suggested ATC12 as the most effective in interfering with the Aurora-A/TPX2 complex formation and Aurora-A activation in mitotic cells (Fig. 3). Therefore, focusing on the latter, we decided to validate its functional effects in breast cancer cell lines (MCF7 and MDA-MB-231) where non-catalytic functions of nuclear Aurora-A have been studied, and based on previous observations that Aurora-A and TPX2 are frequently co-overexpressed in breast cancer [20, 28, 35]. In particular, nuclear Aurora-A promotes transcription of MYC and FOXM1 [35, 38] and a hypoxia transcriptional signature [40], thus favoring migration, invasion, and stemness in TNBC cells. Our results indicate that ATC12 disrupted the Aurora-A/TPX2 complex in both cell lines, highlighting also a decrease in Aurora-A nuclear localization in interphase cells (Fig. 3). This result is in line with our previous observation that Aurora-A overexpression alone is not sufficient to determine its accumulation in interphase nuclei, which is observed only when TPX2 is also co-overexpressed [28], and supports the idea that disrupting the Aurora-A/TPX2 complex may be a good strategy to target protumorigenic nuclear roles of Aurora-A that are independent of its kinase activity.

A decrease in cell viability upon ATC12 treatment was observed in both cell lines through MTT assays (Fig. 4), as similarly reported in Jurkat and HeLa cells using the recently identified CAM2602 Aurora-A/TPX2 interaction inhibitor [25]. Our observation that MCF7 cells are more responsive to the treatment, compared to MDA-MB-231 cells, may reflect different scenarios, including specific cellular backgrounds, activation of specific pathways, or differences in Aurora-A and TPX2 protein levels. Indeed, our observations and data in the literature [41] indicate that while Aurora-A is overexpressed in both cell lines, TPX2 levels are high in MDA-MB-231 but not in MCF7 cells, compared to nontransformed cells. Further investigation will be required to clarify which factors are relevant to yield an effective response to Aurora-A/TPX2 inhibitors.

Dynamic analysis of the effects of ATC12 treatment by time-lapse video recording has also highlighted that, despite a common decrease in dividing cells and, consequently, in the total number of cells, the fate of the MCF7 and MDA-MB-231 cells is different. Induction of death is observed in MCF7 cells, as also confirmed by cleavage of PARP1 (Fig. 5). On the other hand, ATC12 treatment in MDA-MB-231 does not induce cell death, but rather cell senescence, as assessed by induction of p21, increase of nuclear size, and specific assays for β-galactosidase detection (Fig. 6).

Cell death or senescence are stress-inducible states that cells may undergo depending on the severity of the damage or cellular background. It is interesting to note that MCF7 cells display WT p53, while MDA-MB-231 cells harbor a mutated p53 with described suppressive effects on apoptosis [42], which may in part account for the differential response upon ATC12 treatment. Despite the lack of cell death, the observed senescence in MDA-MB-231 cells may have interesting implications for anti-cancer therapy [43–45]. Indeed, induction of senescence by Aurora-A kinase inhibitors has been previously reported [46], and combinatorial treatments with senolytic drugs, to eliminate senescent cells and their potentially adverse induced events, have shown promising synergistic effects [46, 47]. Testing senolytic drugs in combination with ATC12 in MDA-MB-231 cells may therefore represent an interesting future perspective, particularly in light of the need for effective treatments for TNBC. Other combinatorial treatments of potential interest for the therapeutic field are those with canonical anti-mitotic drugs: the combined use of a PPI inhibitor of the Aurora-A/TPX2 complex, analogous to CAM2602, and paclitaxel yields synergic cytotoxic effects on PANC-1 cells, at concentrations that are not effective on cell survival when paclitaxel and the PPI inhibitor are used alone [25]. Interestingly, a study on Diffuse Large B-Cell Lymphoma (DLBCL) also demonstrated that AurkinA synergizes with MLN8237 itself, decreasing MLN8237-induced protumorigenic polyploidy while increasing apoptosis [48].

Taking into account that the use of 3D cultures in breast cancer research has significantly enhanced our understanding of drug resistance mechanisms and the molecular drivers of cancer biology, we also explored the efficacy of ATC12 in mammospheres, a well-known system used for enrichment of cancer stem cells (CSCs), which exhibit high tumorigenicity and chemoresistance features [49–51]. Of note, the relevance of nuclear Aurora-A in promoting the expansion of Breast Cancer Stem Cells has been described [35, 38, 40], and we recently demonstrated that TPX2 co-overexpression promotes Aurora-A-induced MCF10A mammosphere formation [28]. In line with these observations, ATC12 was effective in reducing the size and viability of both MCF7- and MDA-MB-231-derived mammospheres, in contrast to the canonical ATP-competitive inhibitor MLN8237 (Fig. 7), once again confirming the link between Aurora-A oncogenic functions and its interaction with TPX2. Interestingly, the difference in the response to ATC12 between MCF7 and MDA-MB-231 cells is attenuated in the 3D culture system, strengthening the importance of TPX2-regulated nuclear Aurora-A for stemness [28, 35, 38, 40]. Most importantly, ATC12 was effective in TNBC BCOs from patients refractory to cytotoxic chemotherapy, supporting the therapeutic potential of this approach (Fig. 8). The ATC12 concentrations used in our study (medium-high micromolar range) -likely due to the high affinity between Aurora-A and TPX2 and the stability of the complex- and the increased efficacy upon drug renewal in BCOs suggest that further optimizations may yield compounds with higher stability and lower toxicity toward nontransformed cells, with improved potential toward clinical applications. A perspective worth pursuing may also be represented by the use of ATC12 as a starting compound to design Aurora-A-directed PROTACs, which are expected to be effective at lower concentrations with respect to PPI inhibitors. Interestingly, one of the BCOs used in this study responded less efficiently to ATC12 treatment, which, as mentioned above, may result from differences in TPX2 levels or in the genetic background. Further investigation in these directions will help us to clarify the molecular basis of the cellular response to Aurora-A/TPX2 inhibition and predict which tumors may benefit from this strategy.

In conclusion, our results highlight the potential relevance of targeting the Aurora-A/TPX2 complex as a promising target for anti-cancer therapy.

## Materials and methods

### Bioinformatics analysis

The identification of key “hot spots” at the Aurora-A and AurkinA interaction interface was done using the kinase domain structure of Aurora-A (residues 122–403) complexed with the AA35 (AurkinA) compound [PDB ID: 5DN3 [23]]. To examine residue contributions to the stabilization of AurkinA, Computational Alanine Scanning Mutagenesis (ASM) assessed changes in binding energy upon alanine substitution [52].

#### Ligand library preparation

A screening set of 5,806,789 compounds was retrieved from the MolPort database. Conformational sampling was performed using MOE (v.2009; Chemical Computing Group, Montreal, Canada), applying the following parameters: Maximum number of conformers: 25 per compound; Strain energy cutoff: 4.0 kcal/mol (higher-energy conformers discarded); No input filters applied during conformer generation; Default constraint and geometry settings. Compounds were filtered to retain only molecules satisfying typical drug-like constraints: Molecular weight ≤500 Da; Number of rotatable bonds ≤10. This resulted in a total of ~79 × 10^6^ generated conformers.

#### Target structure preparation

The Aurora-A/AA35 complex was edited to remove non-essential heteroatoms, keeping only the AA35 ligand. Protonation states and hydrogen placement were assigned using MOE’s Protonate3D function, which identifies the energetically most favorable protonation pattern. Visual inspection ensured the absence of steric clashes and correct geometry within the binding cleft.

#### Pharmacophore model generation

Pharmacophore modeling was performed in MOE using the Pharmacophore Query (PQ) tool and the Unified Annotation Scheme. Annotation radii for atom-based features were kept at default MOE values (1.0 Å). An exclusion volume with a 4.0 Å radius was generated around residues forming the active site. Given the structural similarity between AA35 and the TPX2 aromatic residues Tyr8 and Tyr10, the aromatic rings of AA35 were marked as essential pharmacophoric elements. The final pharmacophore hypothesis (PH) consisted of the following nine features (coordinates in Å in 5DN3; *r* = annotation radius): HA (35.2, 78.4, –11.4), *r* = 1.0; Hy (36.6, 81.9, –2.6), *r* = 1.0; Hy (31.8, 82.7, –6.7), *r* = 1.0; Ar (34.2, 80.8, –8.7), *r* = 1.0; Ar (37.2, 80.4, –5.5), *r* = 1.0; Ar (32.0, 81.6, –9.2), *r* = 1.0; HA (34.0, 81.3, –7.3), *r* = 1.0; HA (34.3, 80.1, –12.4), *r* = 1.0; NI (34.7, 79.4, –11.7), *r* = 1.0. During screening, compounds were required to match at least three essential features. This filtering reduced the initial library to 73,970 compounds (Supplementary File 1).

#### Docking procedures

Compounds after pharmacophore filtering were docked into the Aurora-A binding cleft using AutoDock Vina [53, 54]. The target structure was optimized using predefined Vina parameters. A grid box centered on the TPX2/AA35 binding interface was generated with the following settings: Grid dimensions: 40 × 40 × 40; Grid spacing: 0.375 Å. Docking simulations employed the Lamarckian Genetic Algorithm, using default values for: Population size; Maximum number of energy evaluations; Mutation rate. The top-scoring poses, defined as those exceeding 2.0 standard deviations above the mean score, resulted in 184 compounds (Supplementary File 2). Among these, 15 compounds underwent final structural refinement and re-docking (Supplementary File 3). These molecules represented the highest-confidence candidates predicted to interfere with the Aurora-A/TPX2 binding interface.

### Protein expression and purification

C290A/C393A Aurora-A double mutant (Aurora-A^KDCM^) cloned in pETM11 vector, was expressed in *E. coli* BL-21(DE3) strain (Novagen-Merck KGaA, Darmstadt, Germany) and purified as previously reported [55]. Fractions containing Aurora-A were collected and dialyzed against 50 mM HEPES pH 7.4, 5 mM MgCl_2_, 300 mM NaCl, 10% glycerol (Buffer A) at 4 °C. For kinase activity assays, Aurora-A was pre-phosphorylated by incubating the kinase with 400 μM ATP (about 10 times the K_M_) for 3 h on ice. To remove the excess of ATP, the protein was dialyzed against Buffer A overnight at 4 °C.

The plasmid for the expression of the 1–43 fragment of TPX2, subcloned in frame with N-terminal GST (kind gift from Richard Bayliss, University of Leeds, UK) was transformed into BL-21 (DE) gold *E. coli* strain (Novagen-Merck KGaA, Darmstadt, Germany); induction with 0.2 mM IPTG (Sigma Aldrich-Merck KGaA, Darmstadt, Germany) was performed overnight at 28 °C. Bacteria were lysed in a buffer composed of 25 mM Tris pH 7.3, 137 mM NaCl, 2.7 mM KCl, 5 mM MgCl_2_, 1 mM DTT, 10% glycerol, supplemented with protease cocktail inhibitors tablets (cOmplete, Roche-Merck KGaA, Darmstadt, Germany). Cell lysis was carried out by sonication on ice (7 min at 70% amplitude in short 10 s pulses with 10 s intervals). Lysate solution was centrifuged at 12,000 × *g* for 30 min, and the supernatant suspension was loaded onto a 10 ml GSH Sepharose 4B (Cytiva-Merck KGaA, Darmstadt, Germany), previously equilibrated with Buffer T (20 mM Tris pH 7.5, 200 mM NaCl, 5 mM MgCl_2_, 10% glycerol). Purified GST-TPX2_1-43_ was eluted with 10 mM GSH (Sigma Aldrich-Merck KGaA, Darmstadt, Germany). Fractions containing GST-TPX2_1-43_ were collected, and concentration was measured in order to add TEV protease in a ratio of 1:100 (protease: GST-TPX2_1-43_) to cleave the GST-tag overnight at 4 °C. Excess GSH was removed with HiPrep 26/10 Desalting column (Cytiva-Merck KGaA, Darmstadt, Germany), and the sample was loaded again onto a GSH Sepharose column. TEV protease was then removed from the flow-through containing TPX2_1-43_.

### Native MS analysis

Protein samples were buffer exchanged into 200 mM ammonium acetate (pH 7.0) through dialysis prior to native MS analyses. These samples were directly introduced into the mass spectrometer using gold-coated capillary needles prepared in-house [56]. Data were collected on a Q Exactive UHMR Hybrid Quadrupole-Orbitrap mass spectrometer (Thermo Fisher Scientific, Waltham, Massachusetts, USA) in positive polarity. The instrument parameters used for MS spectra collection were the following: capillary voltage 1.2 kV, scan range from 1100 to 20,000 m/z, HCD collision energy 0 V, source fragmentation 0 V, and in-source trapping 0 V. The ion transfer optics was set as follows: injection flatapole 5 V, inter-flatapole lens 4 V, bent flatapole 2 V, transfer multipole 0 V. The resolution of the instrument was 8750 at m/z = 200 (transient time of 64 ms), nitrogen pressure in the HCD cell was maintained at approximately 4 × 10^−10^ mbar, and source temperature was kept at 100 °C. The noise level was set at 3 rather than the default value of 4.64. Calibration of the instruments was performed using a 10 mg/ml solution of cesium iodide in water. Data were analyzed using the Xcalibur 3.0 (Thermo Fisher Scientific, Waltham, Massachusetts, USA), NaViA [57], and UniDec [58] software packages.

For TPX2_1-43_ binding experiments: purified Aurora-A^KDCM^ (1 µM) was incubated with TPX2_1-43_ at increasing concentrations (0.125, 0.25, 0.5, 1, 1.5, 2, 3, 4, 6, 8, 12 µM). The sample buffer also contained ammonium acetate (200 mM). Samples were incubated at room temperature for 10 min and were subsequently analyzed by native MS as described above. Peak intensities were then extracted, and the ratios of the intensity of the peptide-bound peak versus the total intensity of all observed species were calculated. Average and s.d. values of these ratio states from three independent experiments were plotted against peptide concentration. The data were fitted globally using GraphPad Prism 8.0 with the equations, in order to account for nonspecific binding events occurring during the native MS analysis:

$${y}_{{specific}}=\frac{{B}_{max}\cdot x}{x+{K}_{D}}$$

$${y}_{{nonspecific}}={NS}\cdot x+{Background}$$

where *x* refers to peptide concentration, *y*_specific_ is the fractional abundance of specific protein-peptide species, y_nonspecific_ is the fractional abundance of nonspecific protein-peptide species, NS is the slope of linear nonspecific binding, expressed in *y* units divided by *x* units, Bmax is the maximum specific binding, in the same units as *y*.

For protein-protein interaction disruption experiments: purified Aurora-A^KDCM^ (1 µM) was incubated with TPX2_1-43_ (1 µM) and each compound at a final concentration of: 1.5625, 3.125, 6.25, 12.5, 25, 50, and 100 µM for ATC4, ATC7, ATC8, ATC12, while the other compounds were only tested at 100 µM. Compound stocks were prepared in 100% DMSO at a concentration of 20 mM; the control consists of 0.5% DMSO. The sample buffer also contained ammonium acetate (200 mM). Samples were incubated at room temperature for 10 min and were subsequently analyzed by native MS as described above. Peak intensities were then extracted, and the ratios of the intensity of the peptide-bound peak versus the total intensity of all observed species were calculated as previously shown [59]. Briefly, average and s.d. values of these ratio states from three independent experiments were plotted against peptide concentration. The data were fitted globally using GraphPad Prism 8.0 with the equation:

$$y\,=B+\,\frac{T-B}{1+\frac{{{IC}}_{50}}{x}}$$

where *x* refers to compound concentration, *y* is the fractional abundance of the Aurora-A/TPX2_1-43_ complex, *B* is the basal mole fraction of the of the Aurora-A/TPX2_1-43_ complex, *T* is the maximal response expressed in terms of Aurora-A/TPX2_1-43_ complex mole fraction, IC_50_ is the concentration of peptide that induces a response halfway between *T* and *B*.

### In vitro Surface Plasmon Resonance experiments

Surface Plasmon Resonance (SPR) was carried out to assess the thermodynamic parameters of the interaction between Aurora-A^KDCM^ (ligand) and three analytes: AurkinA, ATC7, and ATC12, in a Sartorius Octet SF3 apparatus. The ligand was immobilized on an Octet HisCap Sensor Chip, which consists of pre-immobilized nitrilotriacetic acid (NTA) within a nondextran polysaccharide 3D surface, which can be used for affinity capture coupling. The NTA group was activated by nickel ions (Ni^2+^) to bind selectively to the histidine-tagged Aurora-A ligand. The amount of immobilized Aurora-A was detected by mass concentration-dependent changes in the refractive index on the sensor chip surface and corresponded to about 8000 resonance units (RU).

Analytes were dissolved in 100% DMSO at a concentration of 50 mM, and subsequently diluted in sterile 20 mM HEPES pH 7.4, 150 mM NaCl, 0.005% surfactant Tween 20 to yield 2% DMSO final concentration (HSP-2%D buffer) and 200 μM final analyte concentration. Further dilutions and all the experiments were carried out at 25 °C in a degassed HSP-2%D buffer. After ligand immobilization, analytes were injected at different concentrations, i.e., 3.7 μM, 11 μM and 33 μM. During the experiments, based on the Taylor dispersion theory, OneStep gradient injections diffuse single concentrations of analyte into a moving stream of buffer to create a sigmoidal concentration gradient injection of at least 3 orders of magnitude, allowing an accurate measurement of molecule affinity [60, 61]. In all experiments, the increase in RU relative to baseline indicates complex formation between the immobilized Aurora-A ligand and the analytes. The plateau region represents the steady-state phase of the interaction. The decrease in RU after 240 s indicates analyte dissociation from the immobilized Aurora-A after HSP-2%D buffer injection. As a negative control, sensor chips were treated as described above in the absence of immobilized Aurora-A. Values of the plateau signal at steady-state (Req) and full fitting with 1 site were calculated from the overall kinetic evaluation of the sensorgrams using the Octet SPR Analysis software.

### Kinase activity assay

Kinase assays were performed using the ADP-Glo™ Kinase Assay Kit (Promega, Madison, Wisconsin, USA), following the manufacturer’s instructions. Briefly, the reaction mixture (20 μl) containing 100 ng pre-phosphorylated Aurora-A^KDCM^ (see previous paragraphs) and 35 μM ATP, in 16 mM Tris HCl pH 7.5, 8 mM MgCl_2_, 0.04 mg/ml BSA, 0.1 mM DTT, was incubated with different concentrations of each compound at room temperature for 30 min. After incubation, the reaction was started by adding 5 μl of the Myelin Basic Protein (MBP, 1 mg/ml), a substrate of Aurora-A, and was carried out for 30 min at 30 °C. Then, an equal volume (25 μl) of the ADP-Glo™ Reagent was added to terminate the kinase reaction and deplete the remaining ATP. After 40 min, 50 μl of Kinase Detection Reagent was added to convert ADP to ATP and allow the newly synthesized ATP to be measured using a luciferase/luciferin reaction. The produced chemiluminescence, which corresponds to the kinase activity of Aurora-A, was measured as a relative light unit (RLU) with a Luminoskan™ Microplate Luminometer (Thermo Fisher Scientific, Waltham, Massachusetts, USA). Data analysis was carried out with the GraphPad Prism 8 software, using the following equation:

$${\rm{RLU}}=100\times \{1\,-{\left[{\rm{I}}\right]}^{{\rm{n}}}/({\left[{\rm{I}}\right]}^{{\rm{n}}}+{[{{\rm{IC}}}_{50}]}^{{\rm{n}}})\}+{\rm{const}}\,$$

From this equation, we obtained the IC_50_, with [I] as the concentration of inhibitor, *n* the Hill coefficient, and const as residual activity.

### Cell cultures and treatments

MCF-7, MDA-MB-231, as well as MCF10A cells were kindly provided by Prof. D. Barilà (University of Rome “Tor Vergata”) and previously obtained from the American Type Culture Collection (ATCC, Manassas, VA, USA; MCF7 HTB-22, MDA-MB-231 HTB-26, MCF10A CRL-10317). U2OS were from ATCC (HTB-96). Cells were grown at 37 °C and 5% CO_2_ in complete DMEM (U2OS and MCF7 cells) or RPMI (MDA-MB-231) with 10% FBS. Human-nontransformed breast MCF10A cells were grown in HuMEC Basal Serum- Free Medium supplemented with the HuMEC Supplement Kit (12753018 and 12755013, Life Technologies, Thermo Fisher Scientific, Waltham, Massachusetts, USA). All cell lines were routinely tested for mycoplasma contamination.

Treatments with ATC7 and ATC12 (Molport, Riga, Latvia) or MLN8237 (Alisertib; Selleckchem, Houston, Texas, USA) were performed under the indicated conditions. DMSO (0.1%) was used as a control.

For senescence analysis, ATC12-treated cells were incubated for 1 h with 100 nM Bafilomycin A1 (B1793, Sigma Aldrich-Merck KGaA, Darmstadt, Germany) in culture medium to induce lysosomal alkalinization at pH 6 and then for 2 h with 33 μM C_12_FDG (a fluorogenic substrate for β-galactosidase, D2893, Thermo Fisher Scientific, Waltham, Massachusetts, USA).

### Immunofluorescence (IF)

Cells grown on coverslips were fixed by (i) −20 °C methanol, 6 min (ii) or RT, 3.7% formaldehyde plus 30 mM sucrose in PBS for 10 min and permeabilized in PBS containing 0.1% Triton X-100 for 5 min. Blocking and incubations with antibodies were performed in PBS with 0.05% Tween-20 and 3% BSA at room temperature. Primary antibody incubations were carried out for 1 h, except for p21 detection (overnight incubation at +4 °C). Cells were counterstained with 4′,6-diamidino-2-phenylindole (DAPI, 0.1 μg/ml; Sigma Aldrich-Merck KGaA, Darmstadt, Germany) and mounted using Vectashield (Vector Laboratories, Newark, California, USA). Primary antibodies were as follows: rabbit anti-phospho-Aurora-A (Thr288; #3079S, 1:100, Cell Signaling Technology, Danvers, Massachusetts, USA); mouse anti-α-tubulin (T5168, 2 μg/ml, Sigma Aldrich-Merck KGaA, Darmstadt, Germany); mouse anti-Aurora-A (610939, 0.5 μg/ml, BD Transduction Laboratories, Meylan, France); rabbit anti-TPX2 (NB500-179, 1:1500, Novus Biologicals, Cambridge, UK); rabbit anti-p21 (2947, 1:1000, Cell Signaling Technology, Danvers, Massachusetts, USA). Acquisitions were performed with a Nikon Eclipse 90i microscope equipped with the Qicam Fast 1394 CCD camera (QImaging, Surrey, British Columbia, Canada) and 100× (oil immersion; N.A. 1.3) or 40× (N.A. 0.75) objectives, along the z-axis for a total range of (i) 6–8 μm, 0.4 μm z-step (mitosis) or (ii) 5 μm, 0.6 μm z-step (interphases). Acquisitions were performed using NIS-Elements 3.2.

### In situ Proximity Ligation Assays *(is*PLA)

*is*PLAs were performed on cells grown on coverslips and fixed with formaldehyde (see “Immunofluorescence (IF)” section) using the Duolink PLA kit (DUO92007 or DUO92008; Sigma Aldrich-Merck KGaA, Darmstadt, Germany) according to the manufacturer’s instructions. The amplification time has been set to 60 or 80 min to analyze the interaction in mitosis or interphases, respectively. The primary antibody pair to detect the interaction was mouse anti-Aurora-A/rabbit anti-TPX2 (see “Immunofluorescence (IF)” section). In the same reactions, IF staining of the spindle was performed using a chicken anti-α-tubulin antibody (Ab89984, 1:100, Abcam, Cambridge, UK) or anti-α-tubulin FITC-conjugated antibody (F2168, 1:300, Sigma Aldrich-Merck KGaA, Darmstadt, Germany). DNA was stained with DAPI. Acquisitions were performed using a Nikon Eclipse Ti inverted microscope, using a 60× (oil immersion, N.A. 1.4) objective and the Clara camera (ANDOR Technology); or CREST/Nikon V3 Eclipse Ti2 inverted microscope equipped with the Kinetix sCMOS camera (Photometrix) using a 60× (oil immersion, N.A. 1.4) objective, along the z-axis as follows: (i) a total range of 6–8 μm, 0.3–0.4 μm z-step (mitosis) or (ii) 8 μm, 0.6 μm z-step (interphases). Acquisitions were performed using NIS-Elements H.C. 5.02 and NIS-Elements A.R. 5.11.

### Image analysis and quantification

Elaboration and processing were performed using NIS-Elements AR or H.C. (Nikon, Tokyo, Japan) and Adobe Photoshop CS 8.

Signals were measured on maximum intensity projections from acquired z-stacks, after correction for external background. IF and *is*PLA signals were measured at mitotic spindles (Figs. 3A, D, and S3C), at spindle poles (Figs. 3B and S3B), or within nuclei (Figs. 3E, F, and 6E). Values were normalized to the average control (DMSO) value in each experiment. For measures within nuclei, the “general analysis” module of NIS-Elements H.C. was used for automatic recognition based on the DAPI signal in all images. A threshold to automatically identify *is*PLA positive nuclei was set at two standard deviations above the mean of the negative control.

For senescence assays (Fig. 6F and Fig. S4F), fluorescence of whole fields was measured and then normalized for the number of cells within each field.

### Time-lapse video recording

Cells were seeded in 4-well μslides (Ibitreat, 80426, Ibidi, Gräfelfing, Germany) and observed with an inverted microscope (Eclipse Ti, Nikon, Tokyo, Japan) using a 40× DIC objective (N.A. 0.60). During the registration, cells were kept in a microscope incubator (Basic WJ, Okolab, Ottaviano, NA, Italy) at 37 °C in 5% CO_2_. DIC images were acquired every 10 min over 48 h for MCF7 and 72 h for MDA-MB-231 using a Clara camera (ANDOR technology) and the NIS-Elements HC 5.02 software (Nikon, Tokyo, Japan). Movie processing and analysis were performed with Nis-Elements HC 5.02 and Nis-Elements Viewer (Nikon, Tokyo, Japan).

### Western blot (WB)

Cell lysis and WB were performed as previously described [62]. For mitotic shake-off (Fig. S3A), 10 µM S-Trityl-L-cysteine (STLC) (164739, Sigma Aldrich-Merck KGaA, Darmstadt, Germany) was used for 18 h before harvesting. Antibodies were as follows: rabbit anti-TPX2 (NB500-179, 1:1000, Novus Biologicals, Cambridge, UK), mouse anti-Aurora-A (610939, 0.5 μg/ml, BD Transduction Laboratories, Meylan, France), rabbit anti-PARP (9542, 1:1000, Cell Signaling Technology, Danvers, Massachusetts, USA), rabbit anti-phospho-Aurora-A (Thr288)/Aurora B (Thr232)/Aurora C (Thr198) (2914, 1:1000, Cell Signaling Technology, Danvers, Massachusetts, USA), mouse anti-α-Tubulin (T9026, 1:1000, Sigma Aldrich-Merck KGaA, Darmstadt, Germany), and mouse anti-GAPDH (SC-32233, 1:1000, Santa Cruz Biotechnology, Dallas, Texas, USA). HRP-conjugated secondary antibodies (Bio-Rad Laboratories, Watford, UK) were revealed using the Clarity Western ECL Substrate (Bio-Rad Laboratories, Watford, UK). Original data are provided as Supplementary material.

### MTT assay

MCF7, MDA MB-231, and MCF10A cells (2000 for each cell line) were seeded in 96-well plates. After 24 h, 10, 30, 50 μM ATC12 or 0.1% DMSO (as a control) were added for 24, 48, or 72 h. Each dose was tested in technical sextuplicate in each experiment. Then 0.5 mg/ml MTT (3-(4,5-Dimethyl-2-thiazolyl)-2,5-diphenyl-2H-tetrazolium Bromide, Thiazole Blue, 475989, Sigma Aldrich-Merck KGaA, Darmstadt, Germany) was added to each well, and after 4 h of incubation at 37 °C, the formazan salt was dissolved with 200 μl DMSO. The absorbance of each well was measured with the multimode plate reader Clariostar BMG.

### Mammosphere culture, size measurements, and viability assays

Single cell suspensions of MCF7 and MDA-MB-231 breast cancer cell lines were grown in ultra-low attachment 6-well plates (Corning, New York, USA) at a density of 4000 cells/ml in mammosphere medium (Humec 12753018 plus supplement kit 12755013, Thermo Fisher Scientific, Waltham, Massachusetts, USA). After 4 days of mammospheres growth, 50 μM ATC12, 1 μM MLN8237, or 0.1% DMSO were added. After 48 h of treatment, mammospheres were photographed using the ZOE Fluorescent Cell Image System (Bio-Rad, Watford, UK), and the mammospheres' diameters were measured using the ImageJ software (only mammospheres with a diameter >50 μm). For MTS assays, after a week in ultra-low attachment 96-well plates (Corning, New York, USA), formed mammospheres were treated with 30, 50 μM ATC12, 1 μM MLN8237, or 0.1% DMSO. After 72 h of treatment, 20 μl of MTS solution (CellTiter 96 Aqueous One, G3582, Promega, Madison, Wisconsin, USA) was added to each well and incubated at 37 °C for 1–3 h. Finally, optical density (OD) was measured with the multimode plate reader Clariostar BMG, and the survival rates were calculated (% of controls).

### Patient-derived organoids culture and viability assays

Ago-bioptic biopsies were collected from patients treated at “Fondazione Policlinico Universitario A. Gemelli IRCCS” (FPG), Rome, Italy, from May 2020 to October 2024. Protocols were approved by the Institutional Review Board (Protocol ID: 3642) and conducted in accordance with the Helsinki Declaration. All patients enrolled gave their written informed consent for participation. Tissues were processed for organoid development as previously described [36, 37]. For the PDO viability assay, organoids were dissociated with Triple Express and seeded on BME pre-coated 96-well plates in 2% BME growth medium. The day after, PDOs were treated with indicated compounds for 5 days, either continuously or with a compound renewal at half-time of the treatment. Treated PDOs were then collected for cell viability measurement, using the Cell Titer-Glo 3D assay (G9681; Promega, Madison, Wisconsin, USA). PDOs were imaged using the Incucyte platform (10X objective).

### Statistical analyses

Data were statistically analyzed using the InStat3 GraphPad 7: (i) for measurements of continuous variables, unpaired *t*-tests and ordinary one-way ANOVA multiple comparison tests were used; when samples were not normally distributed (assessed by the Shapiro–Wilk normality test), or displayed a variance that was not homogeneously distributed, the Mann–Whitney and Kruskal– Wallis tests were used instead; (ii) for measurements of multiple variables, Two-way ANOVA multiple comparisons test was used; (iii) for measurements of categorical variables, chi-squared (and Fisher’s exact) test in the contingency table analysis was used. All tests are two-sided, and the software recommended that a multiple comparison adjustment be applied where applicable. Outlier filtering was used only when deviation from the expected distribution of the sample population was evident, and assessed by the Identify outliers tools in GraphPad 7 (ROUT method, *Q* = 0.1%; applied to Fig. 3D - MCF7). The number of replicates and sample size are indicated in the corresponding figure legends. No blinded analyses were performed. The criterion for statistical significance (*) was set at *p* < 0.05.

## Supplementary information

Supplementary Figures

Supplementary methods

Table I

Table II

Original WB data

## Data Availability

All data generated in this study are presented in the published article and supported by Supplementary information files. Uncropped western blots are included in the Supplementary material. Raw data are available from the corresponding author upon reasonable request.
