# Supplementary material for: The ATC12 small molecule inhibits the Aurora-A/TPX2 interaction and impairs the proliferation of breast cancer cells
Source: Cell Death Dis. 2026 Mar 24;17(1):356. doi: 10.1038/s41419-026-08579-3 (PMC13039486; doi:10.1038/s41419-026-08579-3)
Supplement: Supplementary file 1 — Supplementary Figures [file 41419_2026_8579_MOESM1_ESM.pdf]

SUPPLEMENTARY INFORMATION

The ATC12 small molecule inhibits the Aurora-A/TPX2 interaction and impairs the proliferation of breast cancer cells

Dalila Boi, Giulia Fianco, Federica Polverino, Francesco Fiorentino, Anna Mastrangelo, Simone Rossi, Elisabetta Rubini, Serena Rosignoli, Francesca Troilo, Maria Rosaria Antonelli, Dalila Tarquini, Laura Cervoni, Serena Rinaldo, Angela Tramonti, Eleonora Kristina Scarpone, Chiara Naro, Claudio Sette, Venturina Stagni, Gianni Colotti, Dante Rotili, Alessandro Paiardini, Giulia Guarguaglini and Italia Anna Asteriti

The PDF file includes: Supplementary figures S1-S5

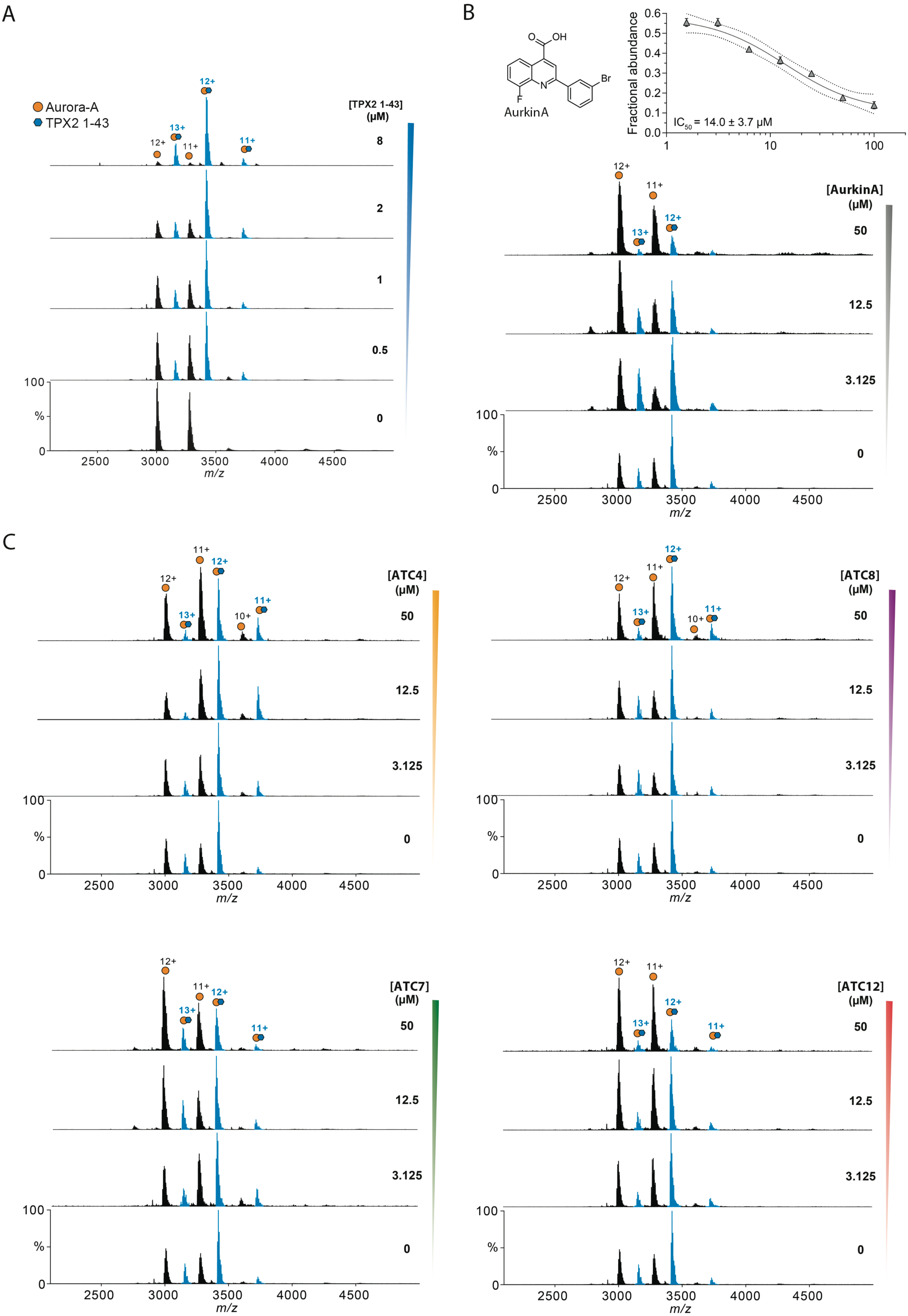

Supplementary Figure 1

Figure S1. Supplementary material related to Figure 1.

A) Native MS analysis of Aurora-A^{KDCM}/$TPX2_{1\text{-}43}$ interaction showing the concentration-dependent increase of peaks corresponding to the Aurora-A^{KDCM}/$TPX2_{1\text{-}43}$ complex. Aurora-A^{KDCM} (1 µM) was incubated with increasing concentrations (0.125 to 12 µM) of $TPX2_{1\text{-}43}$, allowing for the calculation of an apparent K_D. B) Native MS analysis of Aurora-A^{KDCM}/$TPX2_{1\text{-}43}$ interaction in the presence of AurkinA, showing the concentration-dependent decrease of peaks corresponding to the Aurora-A^{KDCM}/$TPX2_{1\text{-}43}$ complex as the concentration of AurkinA increases. The relative plot of $TPX2_{1\text{-}43}$-bound Aurora-A fractional abundance as a function of compound concentration enables the quantification of an IC_{50} value. C) Native MS analysis of Aurora-A^{KDCM}/$TPX2_{1\text{-}43}$ interaction in the presence of ATC4, ATC7, ATC8 and ATC12 showing the concentration-dependent decrease of peaks corresponding to the Aurora-A^{KDCM}/$TPX2_{1\text{-}43}$ complex as the concentration of compound increases. For each assigned complex, the charge state for every peak is shown, with the sign denoting data collection in positive mode.

A

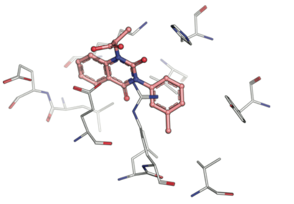

016-908-006 (ATC1)

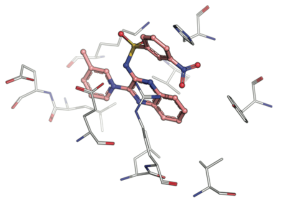

005-907-967 (ATC2)

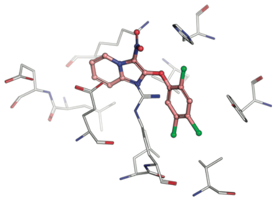

015-381-436 (ATC3)

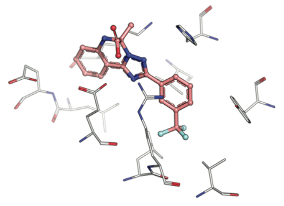

038-995-140 (ATC4)

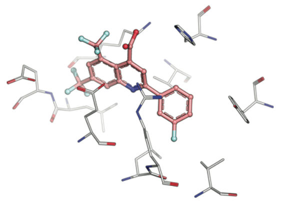

002-918-273 (ATC5)

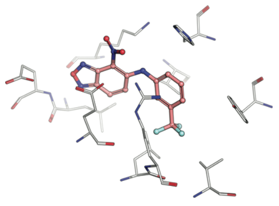

002-112-112 (ATC6)

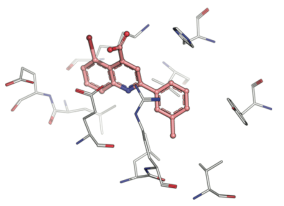

001-574-522 (ATC8)

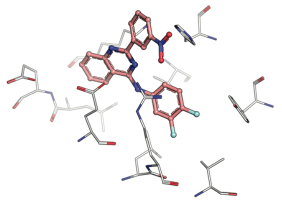

000-698-926 (ATC9)

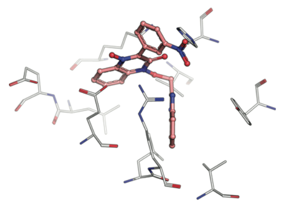

000-819-345 (ATC10)

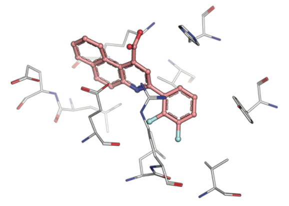

002-709-915 (ATC11)

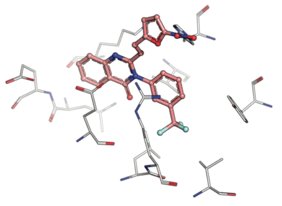

002-956-742 (ATC13)

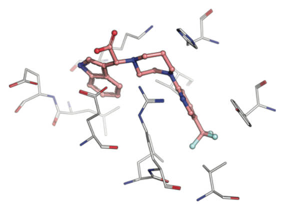

008-320-901 (ATC14)

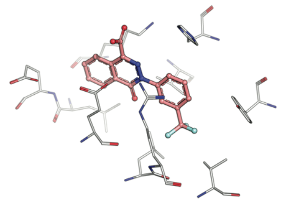

001-778-360 (ATC15)

B

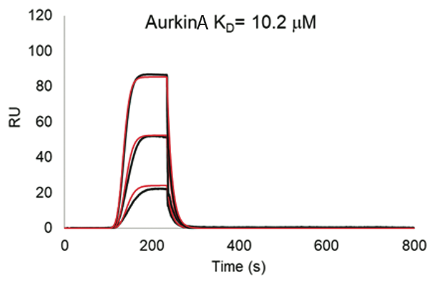

C

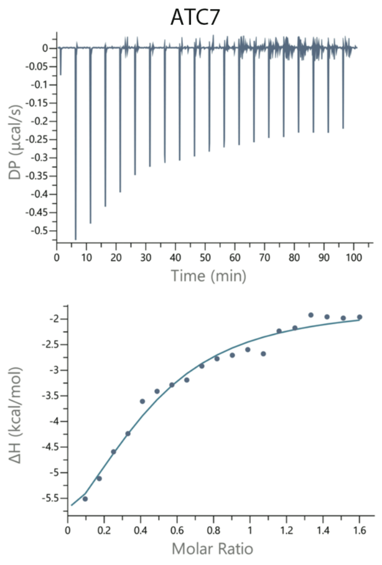

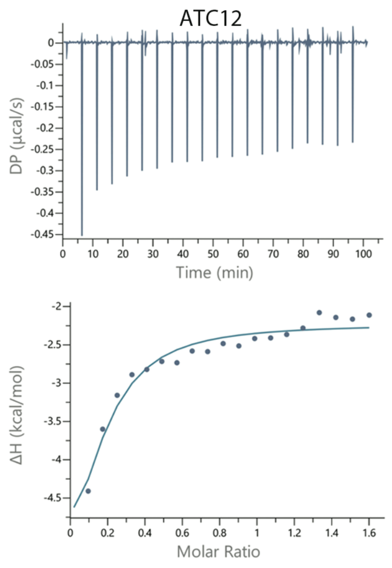

Supplementary Figure 2

Figure S2. Supplementary material related to Figure 2.

A) Top-scored docking poses of the ATC compounds analyzed. ATCs (pink sticks) are shown in their predicted binding conformations within the Y-pocket, for which representative residues are shown in sticks (light grey). B) OneStep sensorgrams showing the interaction of the ligand Aurora-A^{KDCM} with AurkinA under the same conditions described in Figure 2B. C) Binding of ATC7 and ATC12 to Aurora-A^{KDCM} D274N measured by ITC. 38 µM protein solution was titrated with 300 µM of each compound and the titration profile is depicted (upper panels). Integration of the titration peaks produced a sigmoidal enthalpy curve for each interaction (lower panels); a possible heterogeneity of protein population and/or binding site(s) hampered a proper fit with the one-site binding model. Therefore, to get a proper convergent fit, the enthalpy value was set as a fixed value, yielding a K_D=11.1±2.5 µM and a K_D=6.27±0.3 µM for ATC7 and ATC12, respectively.

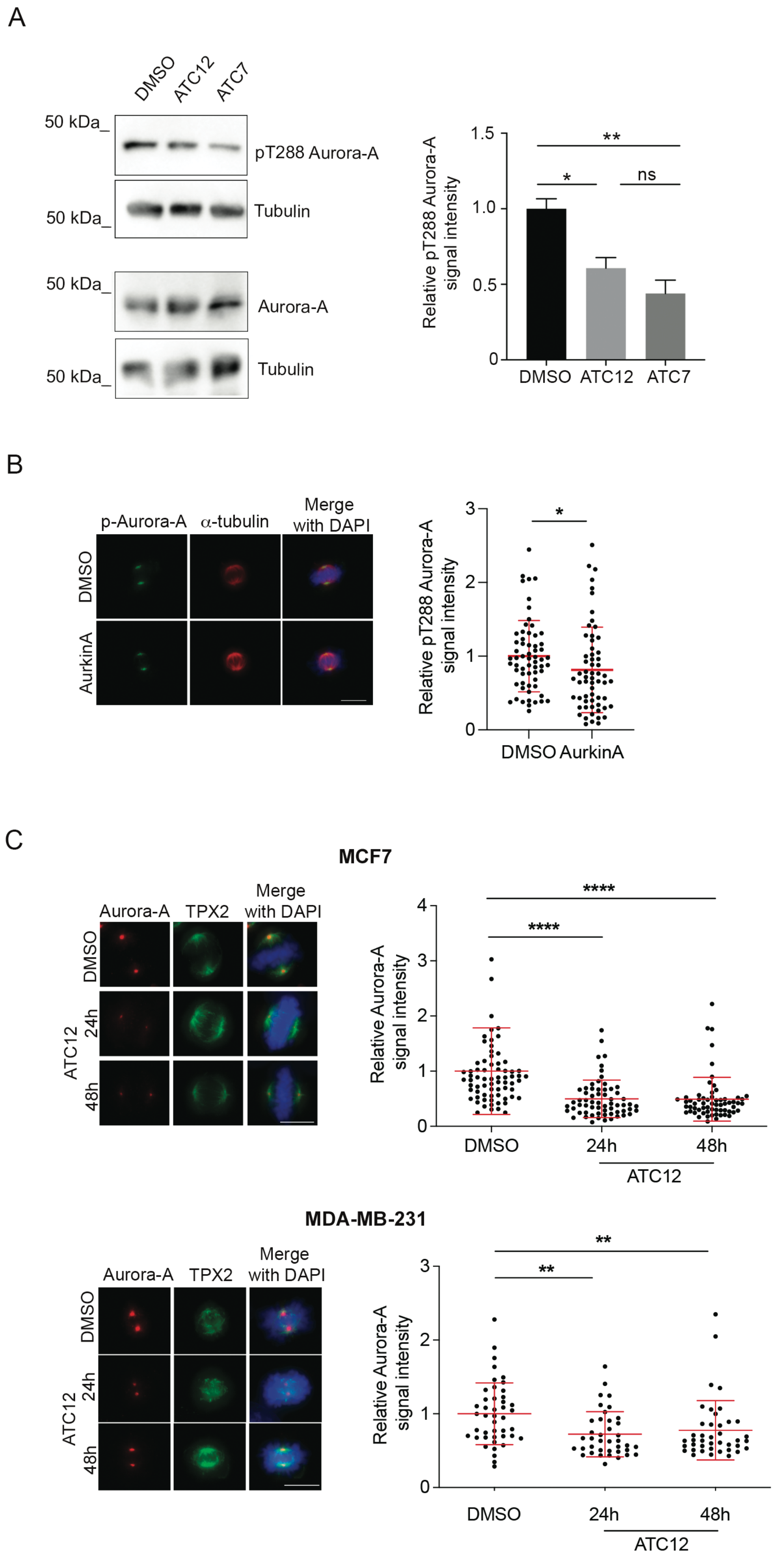

Supplementary Figure 3

Figure S3. Supplementary material related to Figure 3.

A) Representative immunoblotting to detect p-Thr288-Aurora-A (upper panel) and Aurora-A (lower panel) in total lysates from U2OS cells obtained by mitotic shake-off after 24 hours of treatment with 10 µM ATC12 and ATC7, with addition of 10 µM STLC in the last 18 hours. The quantification of p-Thr288 Aurora A signal, normalized to the internal loading control tubulin, is shown in the histogram (n=3). B-C) Representative IF images of p-Thr288-Aurora-A (B, U2OS) or total Aurora-A (C, MCF7 and MDA-MB-231) upon the indicated conditions are shown on the left and quantified in the corresponding dot plots on the right. Sample size per condition: DMSO: 62 (B), 71 (C, MCF7), 44 (C, MDA-MB-231); AurkinA: 61 (B); ATC12 24h: 63 (C, MCF7), 39 (C, MDA-MB-231); ATC12 48h: 63 (C, MCF7), 41 (C, MDA-MB-231). Prometa-metaphase spindles (C) or spindle poles (B) were quantified from two (B; C, MDA-MB-231) or three (C, MCF7) independent experiments. Error bars: SD; *p<0.05; **p<0.01; ****p<0.0001; Unpaired t test (A and B) and Kruskal-Wallis test (C). Scale bars, 10 µm.

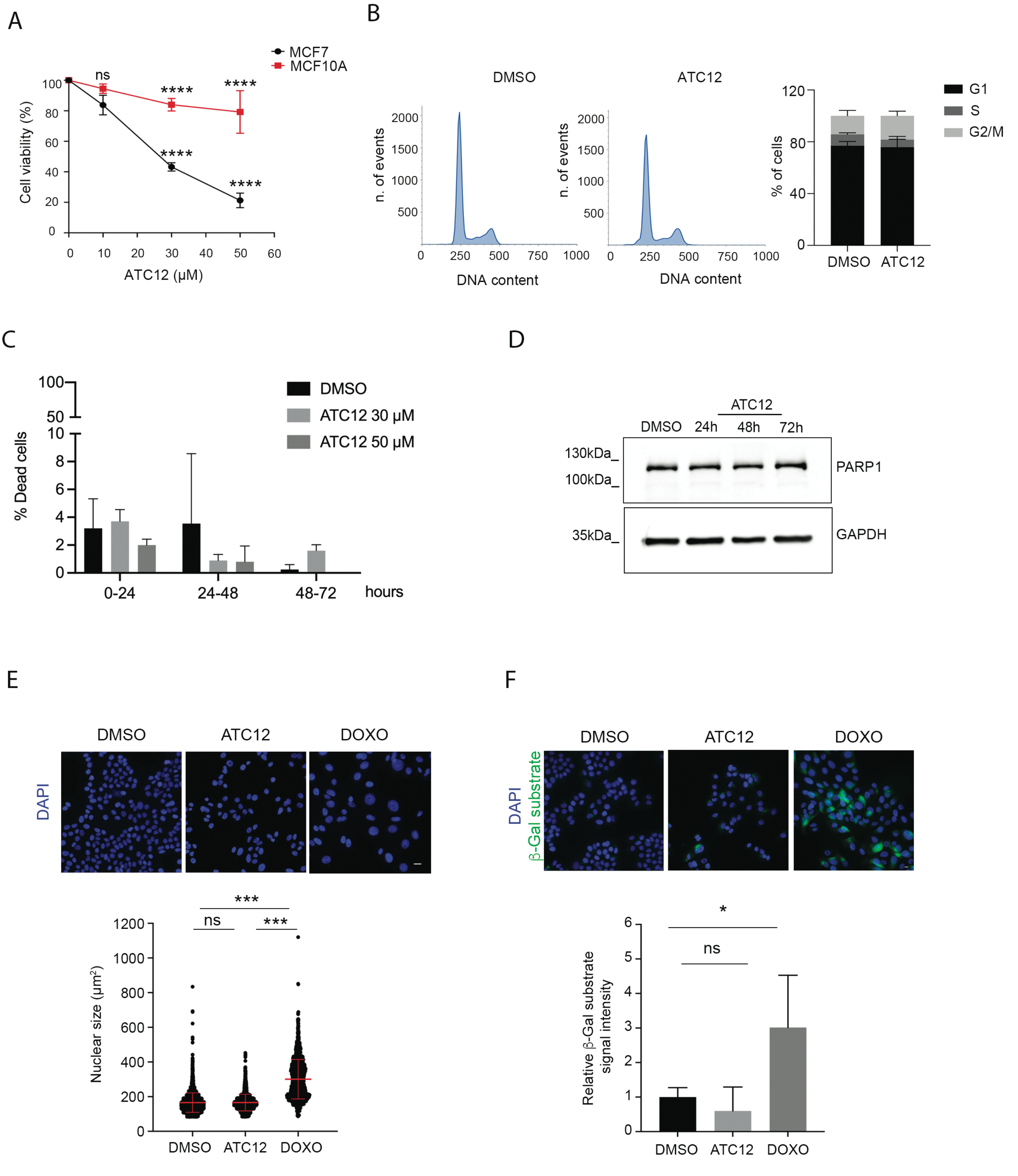

Supplementary Figure 4

Figure S4. Supplementary material related to Figures 5-6.

A) MTT assays of MCF7 and MCF10A cells treated with the indicated concentrations of ATC12 for 48 hours, in technical sextuplicate from four independent experiments. Cell viability decrease is shown with respect to the control condition, set as 100. B) Histograms represent the percentage of MCF7 cells, treated with DMSO or 50 µM ATC12 for 48 hours, in the G1, S and G2-M phases, from three independent experiments. Representative FACS panels under indicated conditions are shown on the left. C) Histograms representing the percentage of dead MDA-MB-231 cells within 24 hours intervals (relative to Figure 6A-D). D) Immunoblotting with PARP antibody of MDA-MB-231 total cell lysates upon 24, 48 or 72 hours treatment with 50 µM ATC12. GAPDH was used as a loading control. E) Representative fluorescence panels of MCF7 cells treated with 50 µM ATC12 for 72 hours. The average nuclear size of ATC12-treated cells (166.06 μm^2) is comparable to that of DMSO (165.37 μm^2). F) Quantification of green fluorescent β-galactosidase substrate (CellEvent™ Senescence Green Detection Kit, C10850, Thermo Fischer, Waltham, Massachusetts). Signal intensity per field is quantified in the graph. The control condition DMSO is set as 1. As a positive control for senescence induction (E, F), parallel cultures were treated with 3 µM doxorubicin (DOXO) for 2 hours, followed by medium renewal and incubation for further 70 hours (until the experimental endpoint). Sample size per condition from three independent experiments: DMSO: 231 (C), 2946 (E), 2892 (F); ATC12 30 µM: 255 (C), ATC12 50 µM: 221 (C), 1705 (E), 1903 (F); Doxorubicin: 1300 (E), 2007 (F). Error bars: SD *p<0 0.05; *** p< 0.001, ****p<0.0001; ns: not significant. Statistical analyses: Ordinary One-way ANOVA multiple comparisons test (A), Fisher's exact test (C), Kruskal-Wallis test (E-F). Scale bars, 10 µm.

A

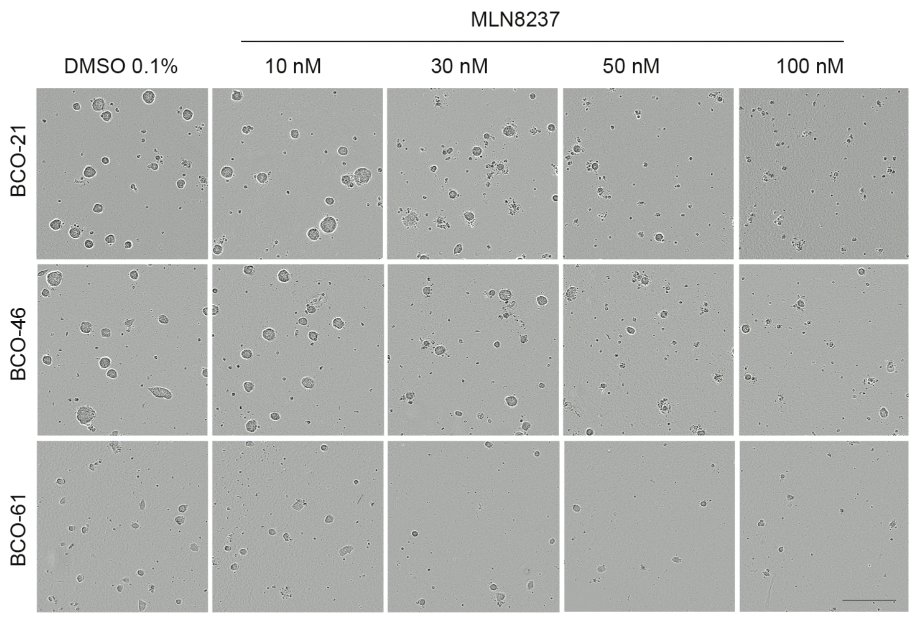
MLN8237
DMSO 0.1%
10 nM
30 nM
50 nM
100 nM
BCO-21
BCO-46
BCO-61

B

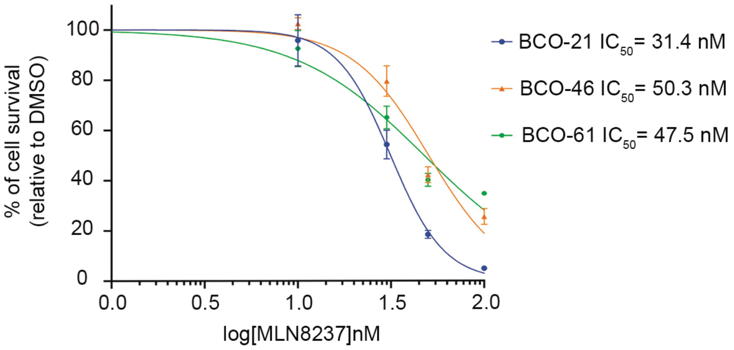
% of cell survival (relative to DMSO)
log[MLN8237]nM
BCO-21 IC50= 31.4 nM
BCO-46 IC50= 50.3 nM
BCO-61 IC50= 47.5 nM

C

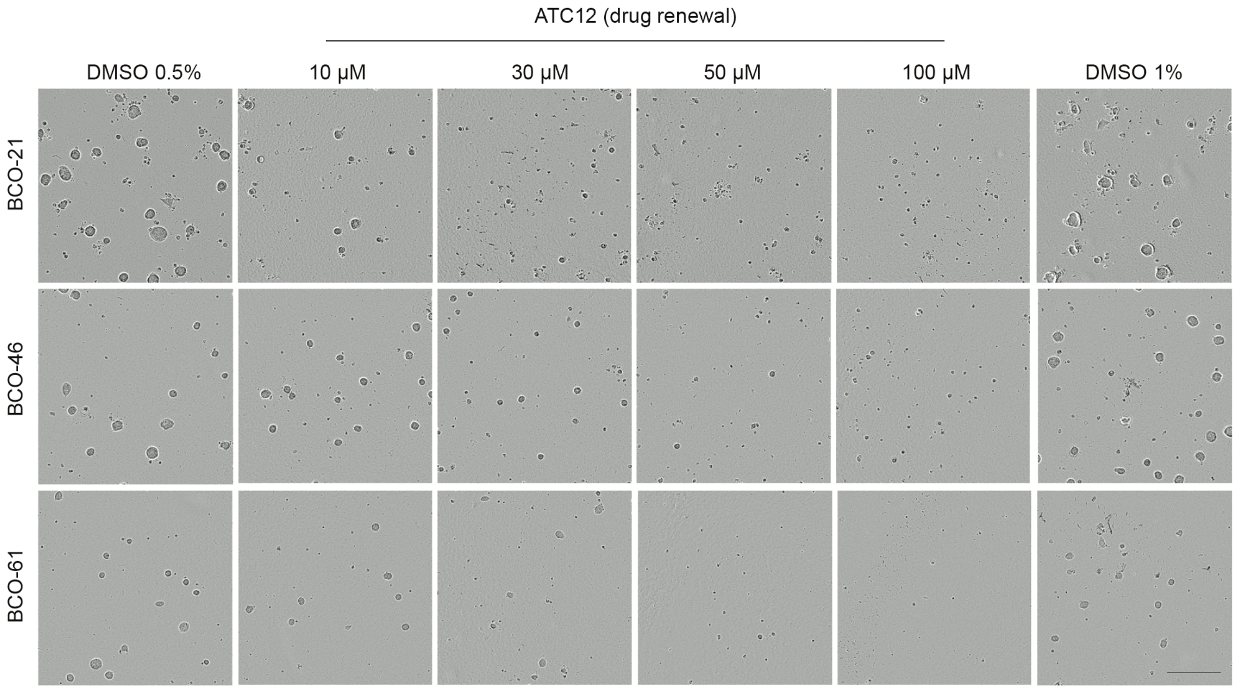
ATC12 (drug renewal)
DMSO 0.5%
10 μM
30 μM
50 μM
100 μM
DMSO 1%
BCO-21
BCO-46
BCO-61

Figure S5. Supplementary material relative to Figure 8.

A) Representative micrographs of BCO-21, BCO-46 and BCO-61 continuously treated with MLN8237 for 5 days, as indicated. B) Dose-response curves of BCO-21, BCO-46 and BCO-61 treated with MLN8237. The fitted curves were used to extrapolate IC_{50} values. C) Representative micrographs of BCO-21, BCO-46 and BCO-61 treated with ATC12 for 5 days, as indicated, with a drug renewal at half time of the treatment. Scale bars, 200 µm.
