# Supplementary material for: The ATC12 small molecule inhibits the Aurora-A/TPX2 interaction and impairs the proliferation of breast cancer cells
Source: Cell Death Dis. 2026 Mar 24;17(1):356. doi: 10.1038/s41419-026-08579-3 (PMC13039486; doi:10.1038/s41419-026-08579-3)
Supplement: Supplementary file 2 — Supplementary methods [file 41419_2026_8579_MOESM2_ESM.pdf]

SUPPLEMENTARY INFORMATION

The ATC12 small molecule inhibits the Aurora-A/TPX2 interaction and impairs the proliferation of breast cancer cells

Dalila Boi, Giulia Fianco, Federica Polverino, Francesco Fiorentino, Anna Mastrangelo, Simone Rossi, Elisabetta Rubini, Serena Rosignoli, Francesca Troilo, Maria Rosaria Antonelli, Dalila Tarquini, Laura Cervoni, Serena Rinaldo, Angela Tramonti, Eleonora Kristina Scarpone, Chiara Naro, Claudio Sette, Venturina Stagni, Gianni Colotti, Dante Rotili, Alessandro Paiardini, Giulia Guarguaglini and Italia Anna Asteriti

The PDF file includes: Supplementary methods

Isothermal Titration Calorimetry (ITC)

For ITC experiments, measurements were carried out with the catalytically inactive mutant Aurora-A^{KDCM} D274N, to keep at minimum sample heterogeneity and ATP/ADP content variability. To this aim, the Aurora-A^{KDCM} plasmid was mutagenized to obtain the D274N mutant. The protein was purified in 50 mM NaHEPES pH 7.5, 500 mM NaCl, 100 mM $(CH_3COO)_2Mg$, 10% glycerol, 0.5 mM TCEP. After elution with increasing concentrations of imidazole, the buffer was exchanged using the PD10 desalting column (Cytiva-Merck KGaA, Darmstadt, Germany) in ITC buffer (50 mM NaHEPES pH 7.5, 200 mM NaCl, 100 mM $(CH_3COO)_2Mg$, 10% glycerol, 0.5 mM TCEP).
A 50 µM protein stock in Buffer ITC was prepared and diluted 1:4 with a 8% DMSO solution, to obtain a 38 µM Aurora-A^{KDCM} D274N solution in 2% DMSO, used as such for the large part of the experiments. Each ligand was dissolved in 100% DMSO and a 50 mM stock solution in 100% DMSO was prepared. A 1.2 mM compound solution was obtained in 8% DMSO and diluted 1:4 with Buffer ITC, to obtain a 300 µM ligand solution to be used for the ITC assays.

ITC experiments were carried out using a MicroCal PEAQ-ITC microcalorimeter (Malvern), with 38 µM of Aurora-A^{KDCM} D274N and 300 µM of ligand both in aforementioned 1x Buffer ITC (see protein expression and purification section for details) and 2% DMSO at 25°C. 2µl aliquots of ligand solution were injected into the protein solution at 25°C (time interval 180 sec, stirring at 750 rpm). Data were fitted using the "one-binding-site model" of the Malvern PEAQ-ITC software. Each condition was assayed at least in duplicate.

FACS

Preparation and analysis of propidium iodide-stained samples was performed as described in Asteriti et al., 2023.
