# Supplementary material for: The ATC12 small molecule inhibits the Aurora-A/TPX2 interaction and impairs the proliferation of breast cancer cells
Source: Cell Death Dis. 2026 Mar 24;17(1):356. doi: 10.1038/s41419-026-08579-3 (PMC13039486; doi:10.1038/s41419-026-08579-3)
Supplement: Supplementary file 3 — Table I [file 41419_2026_8579_MOESM3_ESM.pdf]

Supplementary Table I. Final List of Compounds filtered from Virtual Screening.

2D	MolPort ID	ATC	IUPAC
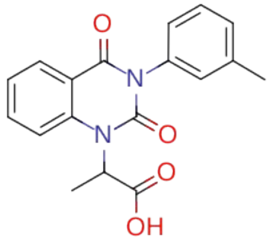	016-908-006	**1**	2-[3-(3-methylphenyl)-2,4-dioxo-1,2,3,4 tetrahydroquinazolin-1-yl]propanoic acid
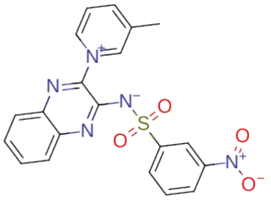	005-907-967	**2**	3-methyl-1-{3-[(3-nitrobenzenesulfonyl)azanidyl]quinoxalin-2-yl}-$1\lambda^{5}$-pyridin-1-ylium
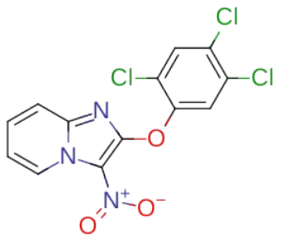	015-381-436	**3**	3-nitro-2-(2,4,5-trichlorophenoxy)imidazo[1,2-a]pyridine
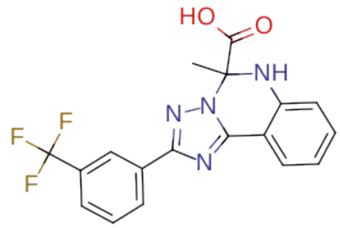	038-995-140	**4**	5-methyl-2-[3-(trifluoromethyl)phenyl]-5H,6H-[1,2,4]triazolo[1,5-c]quinazoline-5-carboxylic acid
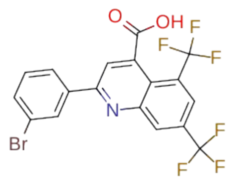	002-918-273	**5**	2-(3-bromophenyl)-5,7-bis(trifluoromethyl)quinoline-4-carboxylic acid

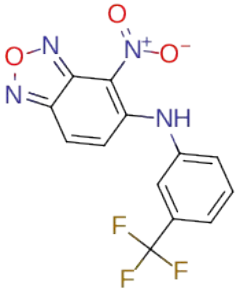	002-112-112	**6**	4-nitro-N-[3-(trifluoromethyl)phenyl]-2,1,3-benzoxadiazol-5-amine
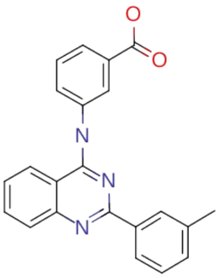	000-664-693	**7**	3-{[2-(3-methylphenyl)quinazolin-4-yl]amino}benzoic acid
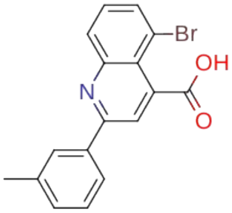	001-574-522	**8**	5-bromo-2-(3-methylphenyl)quinoline-4-carboxylic acid
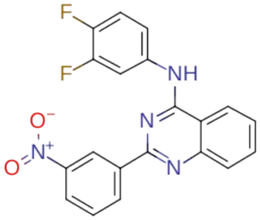	000-698-926	**9**	N-(3,4-difluorophenyl)-2-(3-nitrophenyl)quinazolin-4-amine
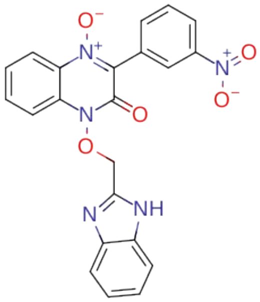	000-819-345	**10**	4-[(1H-1,3-benzodiazol-2-yl)methoxy]-2-(3-nitrophenyl)-3-oxo-3,4-dihydroquinoxalin-1-ium-1-olate

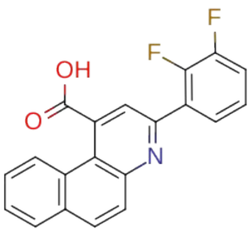	002-709-915	**11**	3-(2,3-difluorophenyl)benzo[f]quinoline-1-carboxylic acid
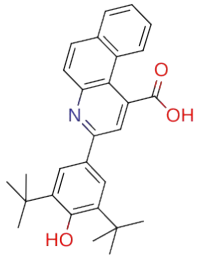	002-709-923	**12**	3-(3,5-di-tert-butyl-4-hydroxyphenyl)benzo[f]quinoline-1-carboxylic acid
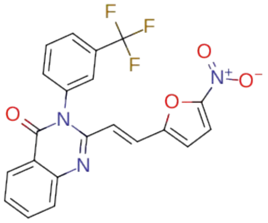	002-956-742	**13**	2-[(1E)-2-(5-nitrofuran-2-yl)ethenyl]-3-[3-(trifluoromethyl)phenyl]-3,4-dihydroquinazolin-4-one
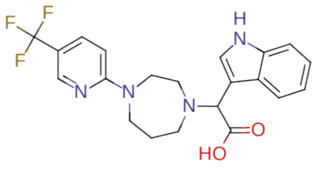	008-320-901	**14**	2-(1H-indol-3-yl)-2-{4-[5-(trifluoromethyl)pyridin-2-yl]-1,4-diazepan-1-yl}acetic acid
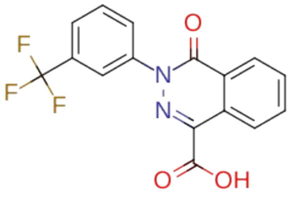	001-778-360	**15**	4-oxo-3-[3-(trifluoromethyl)phenyl]-3,4-dihydrophthalazine-1-carboxylic acid
