# Supplementary material for: The ATC12 small molecule inhibits the Aurora-A/TPX2 interaction and impairs the proliferation of breast cancer cells
Source: Cell Death Dis. 2026 Mar 24;17(1):356. doi: 10.1038/s41419-026-08579-3 (PMC13039486; doi:10.1038/s41419-026-08579-3)
Supplement: Supplementary file 4 — Table II [file 41419_2026_8579_MOESM4_ESM.pdf]

Supplementary Table II: Clinical data of patients from whose pre-NAD biopsies patient-derived organoids (PDOs) were developed.

PDO ID	Age Diagnosis	Cancer type	Subtype	ER	PR	HER2	Ki67	Differentiation Grade	TNM staging	Neoadjuvant therapy (NAD)	NAD response (Tumor residual grade)
BCO-21	49	Ductal invasive carcinoma	TNBC	neg	neg	neg	35	G3	cT4d cN2	Carboplatin/Paclitaxel, Epirubicin-Cyclophosphamide	TRG 4-5
BCO-46	64	High-grade solid carcinoma with pleomorphic features and large areas of necrosis	TNBC	<1	<1	1+	70	G3	cT3 cN3 M0	Carboplatin/Paclitaxel, Epirubicin/Cyclophosphamide +pegG-CSF	TRG 4
BCO-61	64	Ductal invasive carcinoma	TNBC	<1	<1	0	90	NA	cT2 cN0	Carboplatin/Paclitaxel, Epirubicin/Cyclophosphamid, G-CSF, Pembrolizumab	TRG 3
