# Supplementary figures and images for: The ATC12 small molecule inhibits the Aurora-A/TPX2 interaction and impairs the proliferation of breast cancer cells
Source: Cell Death Dis. 2026 Mar 24;17(1):356. doi: 10.1038/s41419-026-08579-3 (PMC13039486; doi:10.1038/s41419-026-08579-3)

Figure 3C

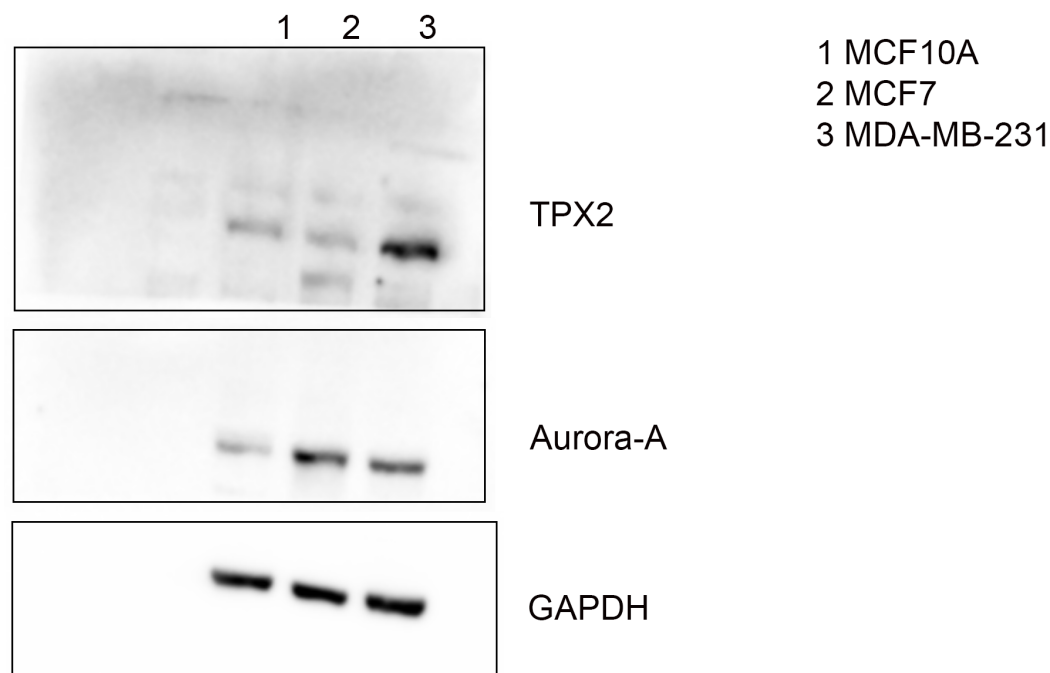

Figure 5F and Supplementary Figure 4D

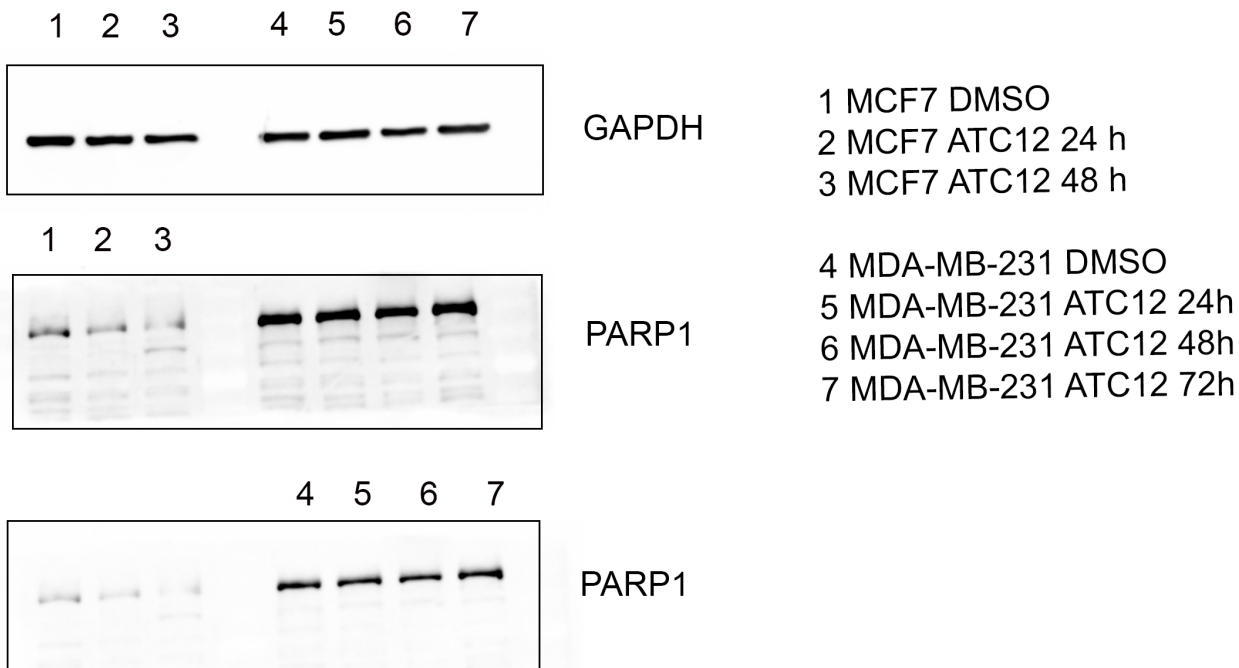

Supplementary figure 3A

1 DMSO  
2 ATC12 10  $\mu$ M  
3 ATC7 10  $\mu$ M

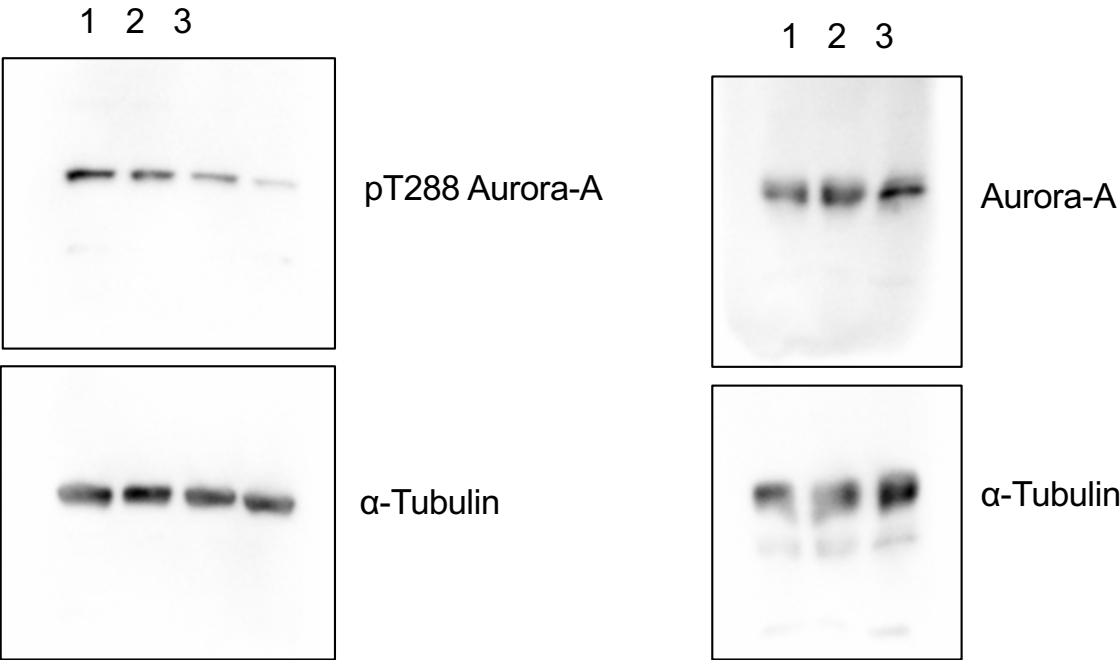

Supplement: Supplementary file 5 — Original WB data [file 41419_2026_8579_MOESM5_ESM.pdf]
